# The structure of a 12-segmented dsRNA reovirus: New insights into capsid stabilization and organization

**DOI:** 10.1371/journal.ppat.1011341

**Published:** 2023-04-21

**Authors:** Qinfen Zhang, Yuanzhu Gao, Matthew L. Baker, Shanshan Liu, Xudong Jia, Haidong Xu, Jianguo He, Jason T. Kaelber, Shaoping Weng, Wen Jiang

**Affiliations:** 1 State key lab for biocontrol, School of Life Sciences, Sun Yat-sen University, Guangzhou, China; 2 Department of Biochemistry and Molecular Biology, Structural Biology Imaging Center, McGovern Medical School at the University of Texas Health Science Center, Houston, Texas, United States of America; 3 Verna and Marrs McLean Department of Biochemistry and Molecular Biology, Baylor College of Medicine, Houston, Texas, United States of America; 4 Institute for Quantitative Biomedicine, Rutgers, The State University of New Jersey, Piscataway, New Jersey, United States of America; 5 Markey Center for Structural Biology, Department of Biological Sciences, Purdue University, West Lafayette, Indiana, United States of America; Indiana University Bloomington, UNITED STATES

## Abstract

Infecting a wide range of hosts, members of *Reovirales* (formerly *Reoviridae)* consist of a genome with different numbers of segmented double stranded RNAs (dsRNA) encapsulated by a proteinaceous shell and carry out genome replication and transcription inside the virion. Several cryo-electron microscopy (cryo-EM) structures of reoviruses with 9, 10 or 11 segmented dsRNA genomes have revealed insights into genome arrangement and transcription. However, the structure and genome arrangement of 12-segmented *Reovirales* members remain poorly understood. Using cryo-EM, we determined the structure of mud crab reovirus (MCRV), a 12-segmented dsRNA virus that is a putative member of *Reovirales* in the non-turreted *Sedoreoviridae* family, to near-atomic resolutions with icosahedral symmetry (3.1 Å) and without imposing icosahedral symmetry (3.4 Å). These structures revealed the organization of the major capsid proteins in two layers: an outer T = 13 layer consisting of VP12 trimers and unique VP11 clamps, and an inner T = 1 layer consisting of VP3 dimers. Additionally, ten RNA dependent RNA polymerases (RdRp) were well resolved just below the VP3 layer but were offset from the 5-fold axes and arranged with *D*_5_ symmetry, which has not previously been seen in other members of *Reovirales*. The N-termini of VP3 were shown to adopt four unique conformations; two of which anchor the RdRps, while the other two conformations are likely involved in genome organization and capsid stability. Taken together, these structures provide a new level of understanding for capsid stabilization and genome organization of segmented dsRNA viruses.

## Author summary

Mud crab reovirus (MCRV), a putative member of the *Sedoreoviridae* family in *Reovirales*, infects an economically important crab, *Scylla serrata*, and has resulted in serious economic loss for the crab aquaculture. In this study, we report the structure of MCRV, which has allowed us to examine the detailed structural elements that might be responsible for capsid stability, as well as those involved in genome packaging and RdRp organization. The arrangement of viral polymerases inside the capsid differ from what has been seen in other viruses of the same order, as ten of the vertices are occupied by RdRp in a symmetrical organization not seen elsewhere. In addition, the VP11 protein is analogous to the “clamp” protein of turreted reovirads.

## Introduction

Infecting a wide range of hosts including marine protists, fungi, plants, insects, crustaceans, fish, birds, and mammals, members of the order *Reovirales* consist of 9–12 segments of dsRNA genome encapsulated by a proteinaceous shell [1]. Infection can result in human diseases and mortality, as well as posing a significant impact to the economy. The order *Reovirales* is divided into two families, *Spinareoviridae* and *Sedoreoviridae*, based on whether they encode for turrets (protrusions or spikes) atop the 5-fold vertices of the virion. The family *Sedoreoviridae*, which includes human pathogens, such as rotaviruses, contains “nonturreted” viruses with 2 or 3 layers of relatively smooth protein shell.

A non-turreted virus consisting of a 12-segment dsRNA genome, mud crab reovirus (MCRV), which may belong to a new order of *Reovirales* [2], infects the economically important crab *Scylla serrata* [2,3]. Like other members of *Reovirales*, MCRV packages its dsRNA segments and a number of RNA-dependent RNA polymerase (RdRp) inside a double-layered icosahedral capsid [2,4]. Underneath an outer T = 13 layer, 120 copies of VP3 form the inner shell; two copies of VP3 in slightly different conformations (termed A and B) are found in each asymmetric unit, and often referred to as a “pseudo *T* = 2” lattice. This organization of the inner shell is conserved not only in the members of *Reovirales*, but also among members of other dsRNA virus families, such as *Totiviridae*, *Partitiviridae*, *Picobirnaviridae*, and *Cystoviridae*; it also bears resemblance to the *Chrysoviridae* structure. For unknown reasons, all but the members of *Birnaviridae and Polymycoviridae* of dsRNA viruses and none of the non-dsRNA viruses have this core arrangement [5–7].

Electron cryo-microscopy (cryo-EM) and X-ray crystallographic structures of several dsRNA viruses, such as mammalian reovirus (MRV) [8] and cytoplasmic polyhedrosis virus (CPV) [9], have indicated a potential role of N-terminal extensions of the VP3-like subunits in connecting inner core subunits, contributing to the stabilization of the capsid. In previous studies of reovirus core particles, large regions of the N-terminus in VP3 homologs were disordered [8,10,11]. It was postulated that the N-terminal regions of the inner capsid proteins might also serve as transcriptional regulating factors for RdRps [12,13].

Across members of *Reovirales*, the RdRps, located on the interior of the inner capsid protein layer, show a conserved finger-palm-thumb core surrounded by N- and C-terminal elaborations, creating a cage-like structure, with four tunnels used for RNA template entry, nucleoside triphosphate (NTP) entry, template exit, or RNA transcript exit [14,15]. Recent reports found ten of the twelve vertices occupied in the 10-segmented CPV [16,17], and eleven of the twelve vertices occupied in the 11-segmented Grass Carp Reovirus (GCRV) [13,18], organized with pseudo-*D*_3_ symmetry (S1 Table). These previously reported results are in agreement with a model where each genome segment is specifically associated with one RdRp [19]. Thus, a 10-segmented CPV and 11-segmented GCRV would package 10 and 11 copies of RdRp, and accordingly, occupy 10 and 11 of the twelve 5-fold vertices, respectively. Interestingly, 10 vertices were occupied by RdRp density, arranged with pseudo-*D*_3_ symmetry, in Fako virus (FAKV) despite having only nine genome segments [20]. Moreover, genome-free particles of *Reovirales* family members still contain the normal number of RdRp [20–23], raising the question of whether capsid and steric constraints collaborate in packaging a set number of RdRps at fixed positions regardless of genomic content. For twelve-segmented dsRNA viruses, like MCRV, the organization of the RdRps inside the capsid remains an outstanding question, which necessitates a structural solution to fill the gap in addressing the packaging and organization of *Reovirales* family members with 9, 10, 11, or 12 dsRNA genome segments.

Here, we present the near-atomic resolution structures of both transcriptionally quiescent MCRV (qMCRV) and actively transcribing MCRV (tMCRV) resolved by single particle cryo-EM with and without icosahedral symmetry imposed. We report distinct conformations for the VP3 molecules clustered around the five-fold pore, suggesting how the N-termini of VP3 stabilize the inner core, as well as how these termini hold the RdRp complex in place and provide a structural framework for regulation of RdRp activity. While previous works have shown that these conformationally variable N-termini can break icosahedral symmetry, this study reveals the organization of these extensions in their asymmetric context, shedding new insights into the interactions that guide the structure and function of the viral protein shell and RdRps. Furthermore, this work clearly defines the presence of ten RdRps with a unique *D*_5_ symmetric organization in the intact virus, despite having twelve dsRNA segments in MCRV. The atomic model of the *in situ* RdRps also provides new molecular insights into capsid interaction and genome organization.

## Results

### Overall structure of qMCRV

**Fig 1:**
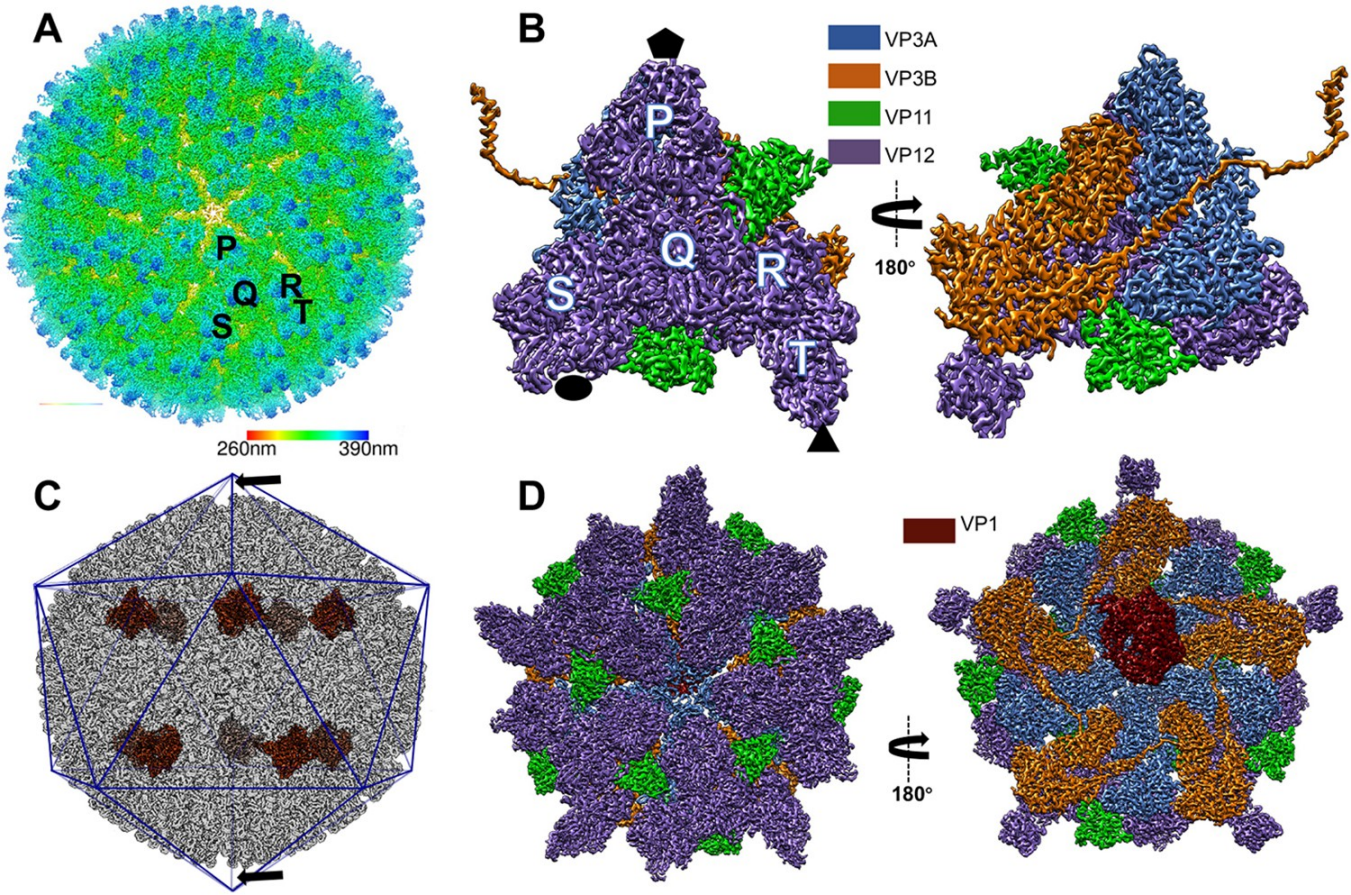
Overall structure of mud crab reovirus (MCRV). (A) A radially colored, shaded surface representation of the MCRV icosahedral reconstruction as viewed along a 5-fold axis is shown. The icosahedral asymmetric unit contains 13 copies of VP12 arranged as 5 trimers: P, Q, R, S and T. Trimer T locates around the icosahedral three-fold axis and thus contributes a monomer to the asymmetric unit. The icosahedral asymmetric unit is shown in (B). (B) The left panel shows an outside view, while the right panel shows the interior view. The inner capsid proteins (VP3A and VP3B), clamp protein (VP11) and outer capsid protein (VP12) are colored in cornflower blue, orange, green and medium purple, respectively. 5-fold, 2-fold and 3-fold axes are indicated by black pentagon, ellipse and triangle respectively. (C) The *D*_*5*_ reconstruction of MCRV indicates that there are 10 well-resolved densities corresponding to VP1 (RdRp) located near 10 vertices and arranged with *D*_*5*_ symmetry. Under the pole vertices (arrows indicated), the density of expected VP1 is too weak to be annotated. VP1 is highlighted in dark red. The densities of other proteins are in grey. (D) The density map of one vertex indicates the arrangements of major capsid proteins in the *D*_*5*_ reconstruction are the same as that of icosahedral reconstruction. The interior view (right) illustrates that the only difference between the icosahedral and *D*_*5*_ reconstructions is the dark-red colored VP1 near the 5-fold vertex. The left panel is an outside view while the right panel is an interior view. The color scheme is the same with that of Fig 1B and 1C.

The three-dimensional structure of the qMCRV was first determined to 3.1 Å resolution with icosahedral symmetry (Figs 1A and S1). The map clearly shows an outer and inner capsid layer; the inner capsid layer is a thin shell composed of 60 copies of a VP3 dimer (Fig 1B), while the outer capsid, arranged on a *T* = 13 lattice, is thicker and composed of VP12 trimers (Fig 1B) organized in a similar way to the previously reported low-resolution reconstruction [3]. Interdigitating these trimers are two smaller densities per asymmetric unit and assigned to be VP11 (Fig 1B). The resolution of the map was sufficient to clearly define the subunit boundaries and protein folds, as well as the majority of the side chain density in each of the protein subunits.

### RdRps are arranged with *D*_5_ symmetry in MCRV

In an attempt to reveal the molecular mechanism of capsid stability, dsRNA genome arrangement and the relationship between the capsid and the genome replication/transcription machines, an asymmetric reconstruction of MCRV was also performed without imposing any symmetry (S2 Fig) [24,25].

Like the icosahedral reconstruction, the capsid proteins in the two layers of the asymmetric reconstruction could be clearly delineated in the density maps. Additionally, densities near the 5-fold vertices and immediately interior to the inner capsid layer were well resolved and assigned as the RdRps (S2 Fig).

Visual inspection of the asymmetric MCRV reconstruction revealed 10 well resolved RdRps around 10 of the 12 icosahedral vertices (henceforth referred as “RdRp vertices”) while the densities around the remaining 2 vertices at the opposite side of the icosahedral center (henceforth referred to as “pole vertices”) were much weaker. To quantitatively evaluate the arrangement of the RdRps, the asymmetric reconstruction was rotated to all 60 icosahedral symmetry related views to position each of the 12 vertices at the north pole in 5 azimuthal views (12×5 = 60 views in total), and the similarities among all possible combinations (60×59/2 = 1770) of the 60 views of the north-pole vertex were computed. The similarity matrix was then subjected to MDS (multi-dimensional scaling) manifold analysis to position all 60 views of the vertices according to their collective similarity distribution. The MDS analysis revealed 6 apparent clusters with each cluster consisting of 10 views of the vertices (S3A Fig). Clusters 1–5 were much tighter when compared to cluster 6, suggesting that the 10 views within each of the first 5 clusters were very similar in structure. The 10 views in each of the first 5 clusters corresponded to the 10”RdRp vertices”, with each vertex contributing 1 of its 5 azimuthal views. Cluster 6 consisted of the 10 views of the 2”pole vertices” with weak densities; its larger spread suggested significant differences in structure among those 10 views. This also suggested that the two”pole vertices” densities in cluster 6 were also distinct from the RdRp densities in the first 5 clusters. If they were not different, these 2 vertices would be structurally similar to the first 5 clusters and included in those clusters, thereby increasing the cluster size from 10 to 12. The large gaps among clusters 1–5 represent significant structural differences between the 5-fold axis related views of the vertex with a single RdRp. Additionally, analysis of the distribution of angles between all pairs (10×9/2 = 45) among the 10 views in each of the 6 clusters (S3B Fig) revealed that the inter-view relationships were quantitively consistent with *D*_5_ symmetry but not other symmetries, including *D*_3_ (S3C Fig).

To further validate the RdRp organization, we employed a decoy mapping method [20], whereby synthetic maps containing RdRps in various configurations (decoys) are used to assay the symmetry in a density map. Here, the decoys consisted of random placements of 8 RdRps (only one RdRp per five-fold axis), a CPV-like configuration of 10 RdRps, a *D*_5_ configuration of 10 RdRps, a *D*_5_ configuration plus one or two polar RdRps, and a *D*_5_ configuration of 10 RdRps where each RdRp is rotated 144°. When compared to these decoys, MCRV particles matched best with the *D*_5_ configuration as discussed above. Particles aligned to the decoys with the *D*_5_ configuration retained the input *D*_5_ configuration; particles aligned to the CPV-like configuration also converged to the same *D*_5_ configuration. When particles were matched to each of the 5 possible positions in decoys that contained the ten equatorial RdRps, they do not appear to match better to any one RdRp position. Moreover, when iteratively refined against these five positions in a multi-model refinement, the RdRps still show no preference to any of the five positions. However, when particles are iteratively refined against a *D*_5_ decoy with one polar RdRp added (total of 11 RdRps), the output structure has the same 11-RdRp configuration as the input structure; no twelfth RdRp appears at the antipodal vertex. As such, the decoy analysis on the MCRV RdRp arrangement is consistent with *D*_5_ symmetry mentioned above. Taken together, these quantitative analyses suggest that our asymmetric reconstruction, including the RdRps, has a unique *D*_5_ symmetry with the 5- and 2-fold axes sharing the corresponding icosahedral axes (S3D Fig), not pseudo-*D*_3_ symmetry as reported in CPV [17], FAKV [20] *etc*.

Furthermore, we analyzed ~3300 “empty” MCRV particles (their genome was absent). After symmetry expansion and 3D classification were performed, most particles were classified into 6 classes with 10 obvious RdRp densities arranged with *D*_5_ symmetry; densities at the remaining two vertices were substantially weaker (S4A and S4B Fig). To quantitate the occupancy at each potential site, we masked out a region of the RdRp that does not overlap with a neighboring 5-fold-related potential RdRp site. For each of the 60 potential RdRp sites, we measured the density enclosed by this mask. The *D*_5_ RdRp sites were over 100 times as intense as neighboring, sterically-occluded sites, but the polar sites were intermediate in intensity: 18% as intense as the 10 highly-occupied sites and 22 times more intense than the unoccupied sites. This is most consistent with additional, polar RdRp choosing randomly among the five-fold-related binding sites, although it remains possible that only 10 RdRp are present and polar density is from misaligned particles, or from other proteins.

The remaining empty MCRV particles were classified into other additional classes, whose densities corresponding to the expected location of the RdRp’s were blurred. Again, we employed a decoy mapping method to validate the RdRp’s arrangement in the empty capsids with RdRps. The decoy set consisted of the closest neighboring 5 RdRps (only one RdRp per five-fold vertex) with random orientations, and the particles matched best with the *D*_5_ configuration once more (S4C Fig).

Imposing *D*_5_ symmetry on the *C*_1_ map preserved the RdRp densities, further verifying the *D*_5_ symmetry arrangement of the RdRps. Subsequent 3D reconstruction with *D*_5_ symmetry imposed resulted in a 3.4 Å resolution map (Figs 1C–1D and S1). All copies of the structural proteins in the *D*_5_ reconstruction were almost identical to those in the corresponding icosahedral map, with the exception of the well resolved ten RdRps near the ten 5-fold vertices (Figs 1C–1D and S1C).

A complete *de novo* atomic model for the MCRV asymmetric unit (2 copies of VP3, 2 copies of VP11, 13 copies of VP12) and RdRp was constructed directly from the icosahedral and *D*_5_ symmetrized maps (S5–S8 Figs and S1 Movie).

### Structure and interactions of the inner capsid protein, VP3

**Fig 2:**
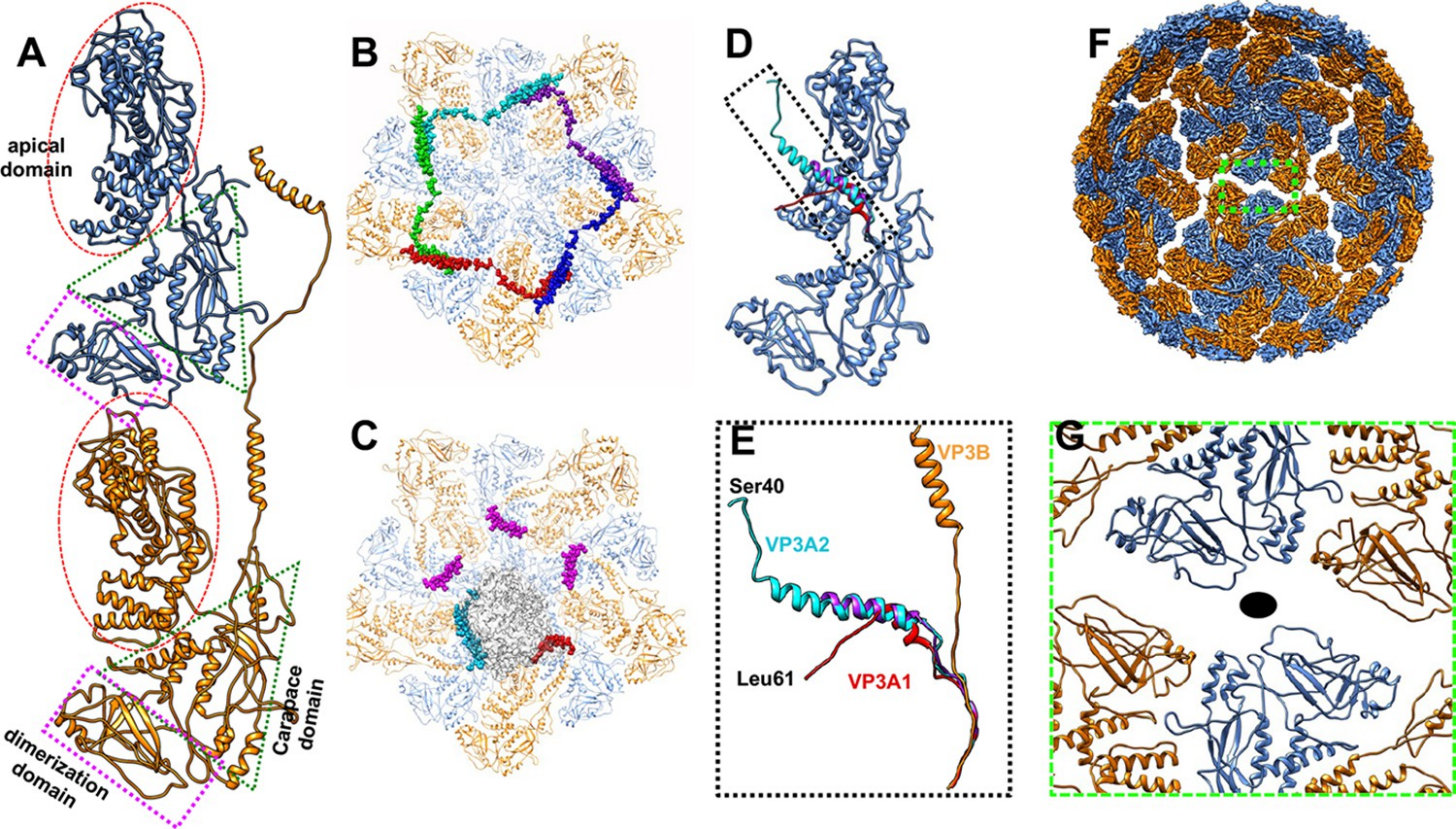
Structure of inner capsid protein VP3. (A) Different conformations of the inner capsid protein VP3A (cornflower blue) and VP3B (orange). Both VP3A and VP3B contain apical (red ellipse), carapace (green triangle) and dimerization domains (purple rectangle). VP3B has an extended N-terminus composed of 2 helices and connecting loops. (B) The five “helix-loop-helix-loop” N-termini of VP3B are indicated using balls and highlighted in different colors. They interact with different VP3 dimers and form a “belt” that stabilizes the inner shell. (C) The N-terminus of VP3A differs from that of VP3B. Three VP3A N-termini, highlighted by purple balls, have similar conformations, while the other two VP3A locations have different conformations (in red and cyan respectively) in the *D*_*5*_ reconstruction. These two N-termini brace the polymerase (gray density) and may help to hold the polymerase to the inner surface of the capsid. The major parts of VP3A and VP3B are colored in cornflower blue and orange respectively, same as that in Fig 1B. The alignment of all VP3A show in (D) indicates that all VP3As are nearly identical, except for the different conformations of the N-terminus. (E) The alignment of the N-terminus of VP3A and VP3B clearly show four different possible N-terminal configurations for VP3. In (F), the large separations among adjacent VP3 pentamers can be seen. (G) A zoomed-in view of the boxed area in (F) shows the large separation at the VP3 dimerization domains. The black oval indicates the 2-fold axis.

A VP3 dimer, containing two chemically identical subunits (A and B) with slightly different conformations in an asymmetric unit, forms the thin inner shell of the MCRV (Figs 1B, 2A and S1C). VP3A and VP3B have nearly identical folds, similar to what is seen in inner capsid proteins of other members of *Reovirales*, forming a flat crescent like structure, divided into three distinct domains (Figs 2A and S5): an apical domain (residues 280–665) composed primarily of α-helices located closest to the 5-fold vertices, a carapace domain (residues 86–279, 666–686 and 819–854), and a dimerization domain (residues 687–818), composed mostly of β-sheets and loops, that interacts with both the neighboring VP3 subunit, as well as VP3 subunits from neighboring asymmetric units.

While the overall fold of the two VP3 subunits are similar, there is considerable difference between the neighboring A and B subunits (~6.9 Å RMSD from residues 86–854). These differences are mainly located in the dimerization and carapace domains (S6 Fig). Specifically, residues 717–750, 760–778, 789–811 at the distal edge of the dimerization domain and residues 258–271 in the carapace domain are shifted by more than 10 Å when the VP3 subunits are aligned (S6 Fig). An additional major structural difference (>10 Å RMSD) is also seen on the inner surface of the carapace domain at residues 608–633 (S6 Fig).

In VP3B, the N-terminus (residues 1–85) consists of a helix-loop-helix-loop motif (Fig 2A) that traverses across the adjacent VP3A, extending towards a neighboring dimer (Fig 2B). Specifically, helix 1 (residues 1–26) of the N-terminus extends across two adjacent VP3 molecules, while helix 2 (residues 54–73) interdigitates the carapace domain of the adjacent VP3A subunit between two α-helices (residues 614–629 and 128–145). The insertion between these two helices likely causes the aforementioned shift in the carapace domain, specifically near residues 608–629. This also likely influences structural rearrangements in the dimerization domain as the α-helix located at residues 128–145 sits at the boundary of the carapace and dimerization domains. With its elongated structure, one VP3B N-terminus interacts with three other VP3 monomers. Residues 39–74 of one VP3B pass through the adjacent VP3A and form an interwoven structure, “locking” the dimer together. Residues 26–38 occupy the middle part of a VP3B molecule from the neighboring dimer, while residues 1–26 insert into the groove of the neighboring dimer’s VP3A. In context, the five N-terminal arms of VP3B form a “belt” around the five-fold vertex that function to link and stabilize the pentamer of VP3 dimers (Fig 2B).

The VP3A N-terminus does not interact with VP3B in the same manner. Rather, based on the *D*_*5*_ reconstructions and corresponding atomic models, it adopts three distinct conformations (Fig 2C–2E). In three of the five symmetry-related VP3A subunits, an α-helix from residues 61–77 bridges the apical and carapace domains, extending towards the adjacent VP3B subunit (Fig 2C). No detectable density is evident in the maps for residues prior to this helix. In one of five VP3A subunits (VP3A1), the aforementioned α-helix becomes shorter (residues 70–81) (Fig 2C–2E). Residues 61–69 form a loop and turn ~30° away, protruding inward and extending down to interact with the RdRp (Fig 2C–2E). The third N-terminal conformation is seen in an adjacent VP3A (VP3A2), where an α-helix extends from residues 50–76 and includes a long loop from residues 40–50 (Fig 2C–2E). It is reasonable to deduce that these varied conformations in VP3A1 and VP3A2 may play roles in maintaining the polymerase position and are further discussed later.

A striking feature of the VP3 shell is the large separation around each 2-fold axis (Fig 2F and 2G). This separation is approximately rhomboidal in shape with sides of 65 Å and 20 Å; this is significantly larger than the corresponding region in other *Reovirales* members. With a gap larger than 8 Å precluding any direct contact, interactions between two dimers of different decamers at the 2-fold axis are exceedingly unlikely. Due to this, it would be nearly impossible for VP3 to maintain stable interactions across two neighboring decamers, and thus there must be additional interactions to bridge neighboring decamers in forming a stable inner capsid (discussed below). As such, the term “dimerization domain” is a misnomer when applied to MCRV VP3.

### Structure and interactions of the major outer capsid protein, VP12

**Fig 3:**
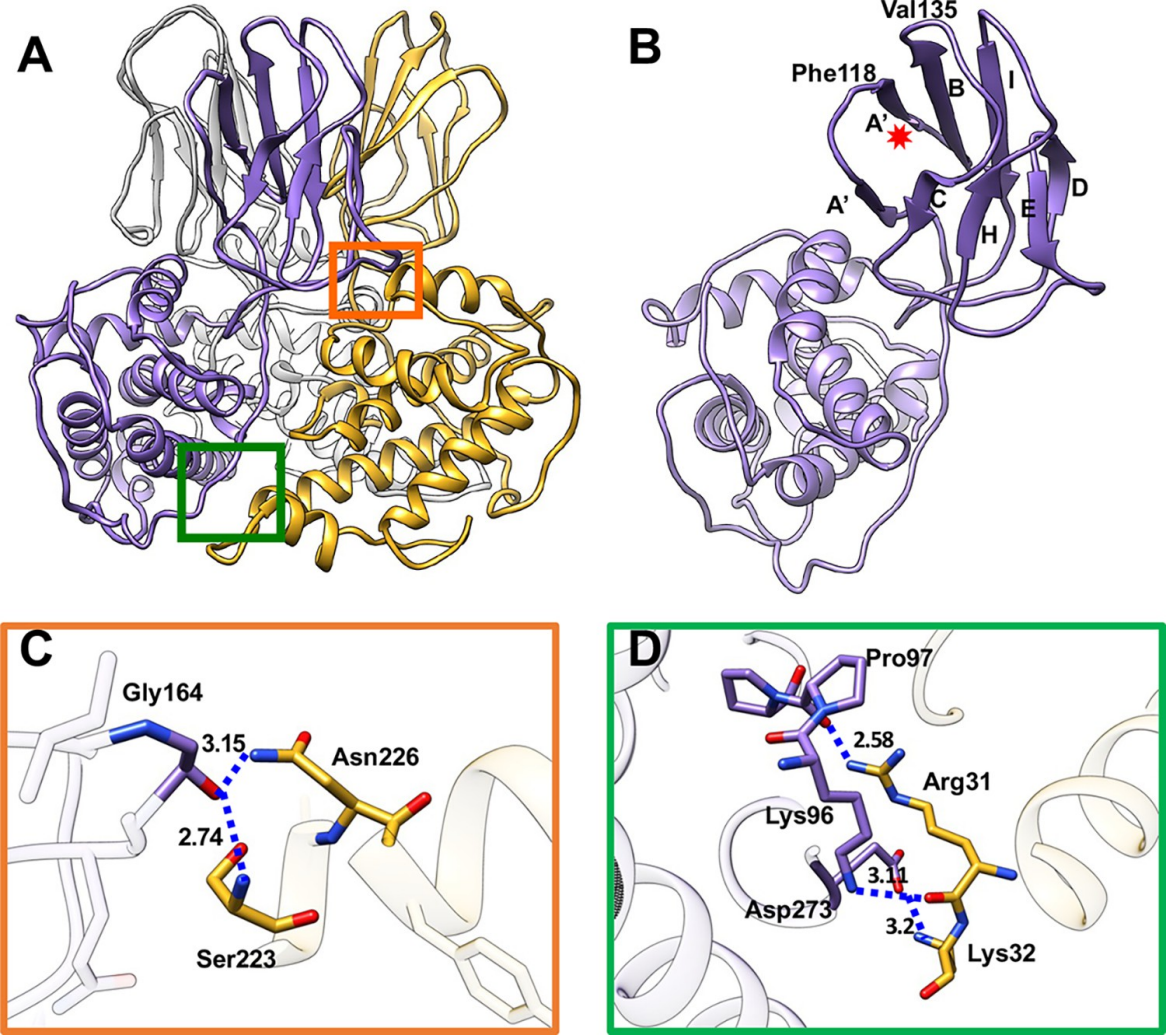
Structure of VP12 and interactions within the VP12 trimers. (A) The model of a VP12 trimer is shown. Three monomers are in medium purple, goldenrod and gray respectively. (B) A single VP12 subunit is annotated. The strand nomenclature is indicated using the upper-case letters A to I. The exterior loops of strands A’A” and BC give rise to a roughly 8Å wide hydrophobic concavity indicated by a red asterisk. (C) A zoomed-in view of the orange boxed area in (A) is shown. Gly164 in one VP12 interacts with the Ser223 and Asn226 of a neighboring VP12 by hydrogen bonds. (D) A zoomed-in view of the green boxed area in (A) illustrates that the PPPG motif in the linker loop interacts with Lys96 of one VP12 and the Arg31 of a neighbor VP12 via hydrogen bonding. There are multiple hydrogen bonds, such as the one between Arg273 and Lys32 of an adjacent VP12.

VP12, the primary outer shell trimer protein (Fig 1B), is comprised of a β-sandwich (two β-sheets of four antiparallel strands) atop an all α-helical base (Figs 3A–3B and S7). In an asymmetric unit of the T = 13 outer capsid layer, VP12 forms 4⅓ unique trimers (Fig 1A and 1B), in which each subunit in the trimer is twisted about the other (Fig 3A). The topology of the β-sandwich domain is virtually identical among MCRV, rotavirus [26], rice dwarf virus [11], bluetongue virus [10] and African horse sickness virus [27], though spinareoviruses, such as aquareovirus (ARV) [28], differ considerably. MCRV VP12’s β-sandwich domain is incomplete, with strands A”BID and A’CHE as opposed to A”BIDG and A’CHEF in rotavirus [26] (Fig 3B). Interestingly, all four loops of the MCRV β-sandwich are found in representative sedoreoviruses (A’A”, BC, DE, and HI in the rotavirus/bluetongue virus nomenclature) [26,29]. The A’A” and BC exterior loops give rise to a hydrophobic concavity of roughly 8 Å in width, opening to Phe118 and Val135 (Fig 3B). The position of this pocket at the outer extreme of the capsid radius, as well as its shape and hydrophobicity, might relate to cellular interactions with MCRV; however, this needs further investigation.

In the outer capsid, a major contributor to intra-trimer interactions can be found at the edges of the β-sandwich in the VP12 trimers, where a loop containing a di-glycine is stabilized by the hydrogen bonding of Gly164 with Ser223 and/or Asn226 of a neighboring VP12 (Fig 3C). A second interaction between neighboring VP12 trimers is mediated by linkers connecting the α-helical base to the β-sandwich (Fig 3D). Each of residues 92–102 is within 5 Å to the neighboring trimer; largely polar contacts are seen between sidechain and mainchain atoms. As an example, the Lys96 amine group is positioned to form a hydrogen bond with the carbonyl group of Arg31 in the neighboring trimer, whose amine group reaches back to interact with Pro97 in a stretch of three consecutive prolines (Pro97-Pro98-Pro99) (Fig 3D). The PPPGF motif in this linker can assume α-helical (i.e. PDB-4OZV [30]), turn (i.e. PDB-5C33 [31]), or polyproline-II conformations (i.e. PDB-4P45 [32]) depending on the structural context [33]. While several other members of *Reovirales* VP12 homologs also have two or more prolines in this linker region, only MCRV contains a polyproline-II motif here. This secondary structure is known to render the backbone more solvent-exposed, which may be related to the extensive mainchain involvement in trimer-trimer interactions.

### Structure and interactions of the outer capsid protein VP11

**Fig 4:**
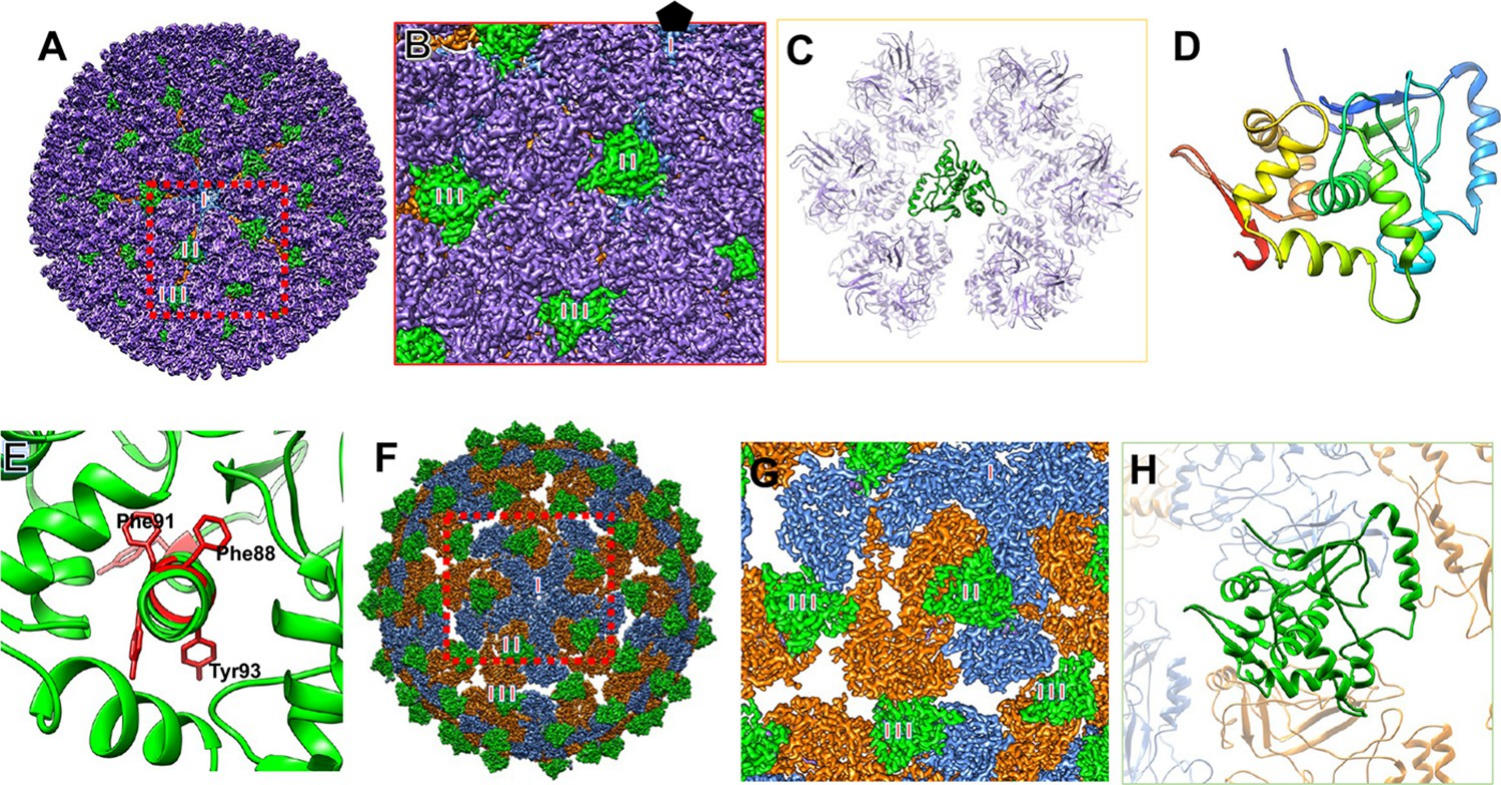
Locations and structure of VP11 implies that it may function as a clamp protein in MCRV. (A) The outside view of MCRV capsidis shown. In (B), a zoomed-in view of the boxed regions in (A) can be seen; relative orientation of this view can be inferred based on the indicated location of the 5-fold vertex. Type I, II and III channels are labeled and show the potential aqueous channel positions. The color scheme is the same as that of Fig 1B. VP11 (green) not only inter-digitates the VP12 trimers, the two copies of VP11s in an asymmetric unit also block type II (VP11A) and III (VP11B) channels. (C) VP11 sits in the middle of the six VP12 trimers and interacts with the 6 VP12 trimers in different manners, clamping these trimers together. (D) The atomic model of VP11 is shown in rainbow color. The colors from blue to red indicate the N-terminal to C-terminal. (E) VP11 has a core hydrophobic α-helix (L82-Y98) with five aromatic residues (red). Panel (F) shows the same view in (A) after removing all VP12 trimers. Panel (G) shows a zoomed-in view of the boxed region in (F). VP11 rests on top of VP3 dimers from the inner capsid. (H) VP11B is located on the top of the separation between VP3 dimers from adjacent pentamers, interacting with VP3A from one pentamer and VP3B from the other pentamer. Through these interactions, VP11B clamps the two neighbor pentamers together. The color schemes are the same as that in Fig 1B, except for that in (D).

Unique to MCRV, the outer capsid layer contains a second capsid protein, VP11 (S8A Fig). Two VP11 subunits per asymmetric unit are embedded between VP12 trimers and bridge multiple asymmetric units (Figs 1B and 4). One of the two VP11 subunits (VP11A) bridges the adjacent asymmetric units around the same 5-fold vertex and block the narrow opening, previously denoted as a type II channel [34], while the other (VP11B) is located along the icosahedral 2-fold axis and block the type III channel (Fig 4A and 4B). In both cases, each VP11 is bound by six VP12 trimers (Fig 4C), three from each asymmetric unit, at very different interfaces. The two VP11 subunits are almost identical (RMSD ~0.79Å over 203 residues), having some slight conformation difference localized to the peripheral loops and termini, likely a result of their different environments (S8B Fig).

VP11 has an overall tetrahedral shape, consisting mostly of α-helices and two small β-sheets (Figs 4D, S8A and S8C). Additionally, VP11 contains a core hydrophobic α-helix (Leu82-Tyr98) with five aromatic residues (Phe88, 91; Tyr93, 94 and 98) and five leucines (Leu86, 87, 89 and 95), around which several other helices are wrapped (Fig 4E). The two small β-sheets serve as exterior elaborations interacting with the VP12 trimers.

Sequence and structural searches revealed no VP11 structural homologs, indicating that this protein contains a novel fold. While the exact function of VP11 remains to be confirmed, some parallels can be drawn to other *Reovirales* family members in an attempt to infer the role of VP11. In other members of *Reovirales* with clamps, the analogous VP11 positions are largely occupied by clamp proteins that appear to engage many residues of neighboring VP12-like trimers. In λ1, the clamp binds at three distinct locations within each icosahedral asymmetric unit [8] through interactions that are broadly conserved among spinareoviruses. As such, VP11 may in fact be a clamp protein, despite the lack of detectable sequence or structural similarity to other viral clamp proteins.

There is an abundance of hydrophobic amino acids at the interface between VP11 and VP12, resulting in a number of potential hydrophobic interactions (S9A and S9C Fig). In addition, several hydrogen bonds are also found at the VP11/VP12 interface (S9B Fig). The interface area between VP11 and six VP12 is about 2,472 Å^2^. Through these extensive interactions, VP11A may “clamp” the six adjacent VP12 trimers together. VP11A also interacts with the inner capsid protein, VP3. A group of polar amino acids are seen at the interface between VP11A and VP3 (S9D and S9F Fig), as well as several potential hydrogen bonds (S9E Fig).

A striking feature of MCRV is the large separation in the inner capsid underneath VP11B at the type III channel as mentioned above (Fig 4F and 4G). VP11B may provide the “missing” interactions to clamp the neighboring VP3 inner capsid proteins that have no direct interactions among different decamers. The two ends of VP11B straddling this separation interact with the equivalent sites in the dimerization domain (an α-helix at residues 734–744 and a loop at 696–702) of VP3 (Figs 4H and S9G), even interacting with some of the same VP3 residues (Asn697 and Asp699) (S9I Fig). These interactions are mediated by hydrophobic interactions and hydrogen bonds at the interfaces between VP11B and VP3B (S9H–S9J Fig). Together, the extensive interactions between VP12 and VP3 dimers, as well as VP11’s interactions with VP12 likely stabilize the inner VP3 layer despite the large separation in subunits.

Both the clamp proteins of GCRV [35] and CPV [9] engage the same elements of the dimerization domain, but the interacting regions of the clamp involved show little resemblance to that of MCRV VP11. The clamps of GCRV do not have interactions with the outer capsid. As genes typically evolve with higher conservation of fold topology than protein-protein interactions, the history of this orthologous set is unusual and may reflect the unique demands of viral capsids.

### Structure of VP1, the RdRp

A hallmark of the MCRV non-icosahedral (*C*_1_ and *D*_5_) (S2 and S3 Figs) reconstruction is the ten well-resolved RdRp densities located slightly offset (~30 Å) from ten of the twelve icosahedral vertices that are organized with *D*_5_ symmetry (Fig 1C and 1D). Along the remaining two icosahedral vertices (coincidental with the 5-fold axis), a small amount of density is seen at significantly lower thresholds. As with the other protein subunits in the MCRV reconstructions, it was possible to construct atomistic models for the RdRp.

**Fig 5:**
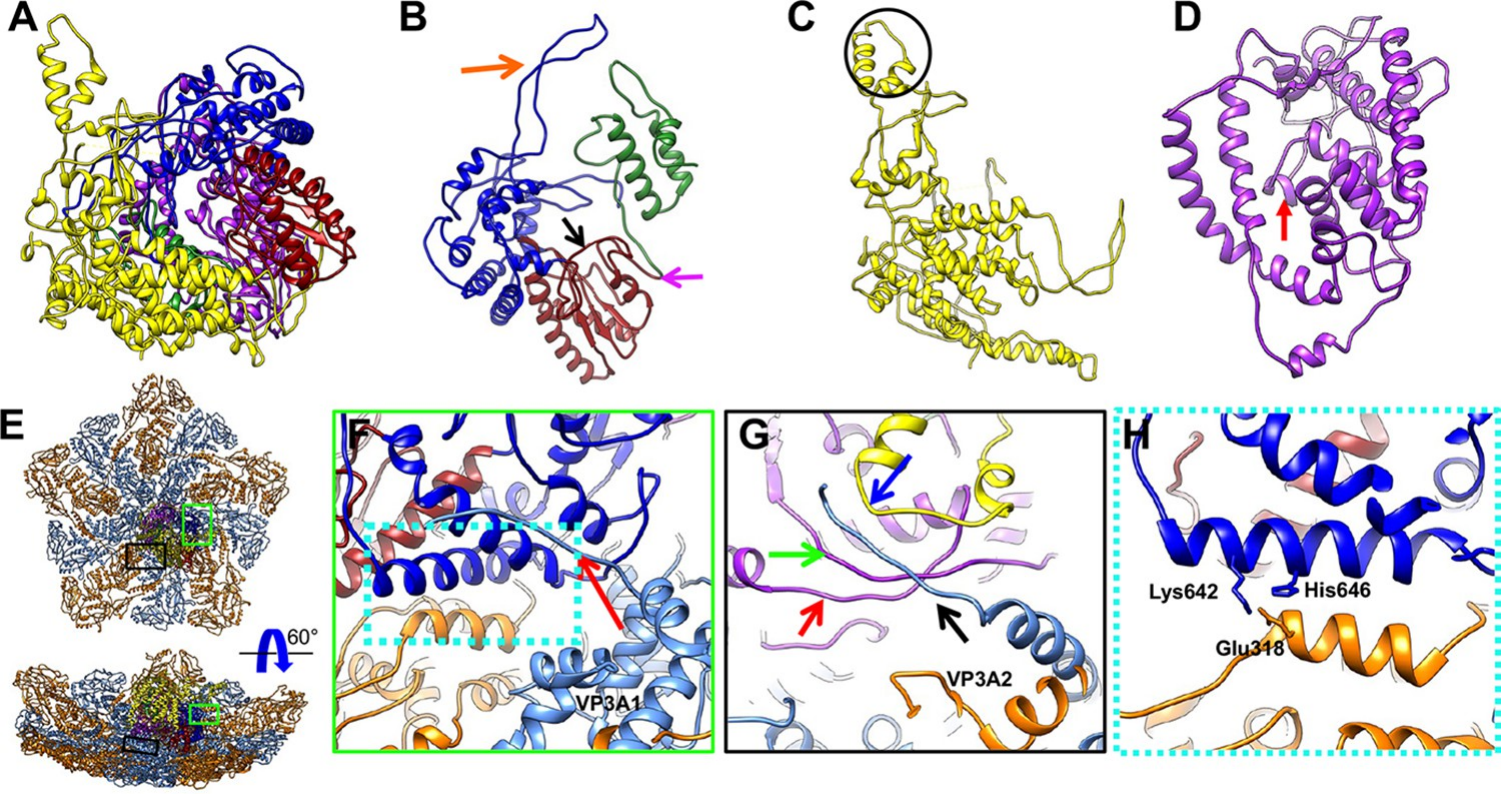
Structure of VP1 (RdRp). (A) The model of the RNA dependent RNA polymerase (RdRp), VP1, is shown. Like other Reovirus polymerases, VP1 contains an N-domain (yellow), C-domain (purple) and polymerase domain. The polymerase domain is further divided into the finger subdomain (blue), palm subdomain (dark red) and thumb subdomain (green). The Polymerase domain of the RdRp is shown in (B). The purple arrow indicates the primer grip, the black arrow indicates the priming loop and the orange arrow indicates the unique long loop blocking the gap between the thumb and finger subdomain. (C) The N-domain has a unique protrusion (black circle). (D) The C-domain is shown. The red arrow indicates the C-terminal plug. The overall view of the inner VP3 layer and the RdRp protein VP1 is shown in (E), while (F) shows a zoomed-in view of the green box in E. The VP3A1 N termini (residues 59–69) (red arrow) interacts with the finger subdomain of the RdRp VP1 (blue). (G) A zoomed-in view of the black box area in E is shown. N-terminal residues (40–48, black arrow) of VP3A2, and residues 1220–1226 (green arrow), 67–74 (blue arrow) and 1390–1395 (red arrow) of VP1 form an anti-parallel β-sheet. (H) The zoomed-in view of the cyan-dashed box in F demonstrates the interactions between the apical domain of VP3B and the VP1. Residues 318–346 of VP3B apical domain (orange) form a helix-loop-helix structure along the inner surface and interact with residues 642–657 (blue) of finger subdomain of an RdRp. Residues Lys642 and His646 in the RdRp appear to form a salt bridge with Glu318 of VP3B.

The structure of the RdRp revealed a compact, cage-like structure ~75 Å in diameter with an extended protrusion near its innermost surface (Fig 5A). This cage-like structure is a common feature in RdRps seen in λ_3_ of reovirus [15], VP1 of rotavirus [12,14,36], and the RdRps of cypovirus [16,17], aquareovirus [13,18] and bluetongue virus [37] (S10 Fig). The MCRV RdRp can be divided into three domains: an N-terminal domain (residues 1–617), a central polymerase domain (residues 618–1090), and a C-terminal bracelet domain (residues 1091–1422) (Fig 5). The central polymerase domain can be further divided into three sub-domains: a finger sub-domain including residues 618–775 and 823–896, a palm sub-domain composed of residues 776–822 and 897–1008, and a thumb subdomain that contains residues 1009–1090 (Fig 5B). As with other reovirus RdRps, the arrangement of these domains forms a relatively hollow center that is presumably the catalytic center of the RdRp.

As in all polymerases, the central polymerase domain contains the core catalytic residues. In particular, D819, D824, D947 and D948 form the conserved acidic residues in the active site and residues 1003–1012 form the “primer grip” which was thought to be close to the phosphate that joins the nucleotides at the primer terminus [38] (Fig 5B). Also, the priming loop, residues 776–796, is seen in the ‘down’ conformation in our MCRV structure (S10 Fig). In rotavirus VP1, the down conformation is incapable of supporting initiation of transcription in the priming site [14]. Also like rotavirus VP1, the finger sub-domain lacks a four-stranded β-sheet which protrudes from the surface of the λ3 of reovirus. However, a long loop from residues 675–719 in the MCRV blocks the gap between the thumb and finger subdomains (Figs 5B and S10), a feature unique to the MCRV RdRp.

The N-terminal domain is mainly helical and forms a “closed” continuous surface on one side of the RdRp that bridges the finger and thumb sub-domains (Fig 5C). This surface is almost perpendicular to the inner capsid layer though only two small stretches of residues in this domain (67–74 and 523–545) appear to interact with a single VP3A. A protrusion on the innermost surface of the RdRp (residues 324–374) extends outward, which is a unique feature not found in other *Reovirales* polymerases (Figs 5C and S10). This protrusion, along with another small portion of the N-terminal domain and finger subdomain forms an open tunnel (S11A Fig), which is likely the RNA template entry tunnel [15]. Nearby is another tunnel, similar to the reported NTP entry tunnel [15], composed of two helices (556–574 and 269–279) and a small β-sheet from this N-terminal domain along with the palm and finger subdomain (S11A Fig).

Like the N-terminal domain, the C-terminal domain is composed primarily of α-helices and bridges the thumb and finger subdomains on the opposite side of the N-terminal domain (Fig 5D). This domain is similar to those reported in other reovirus polymerase structures [14]. In λ3 and rotavirus VP1, the C-terminal domain encircles the dsRNA and -RNA template exit tunnel. Likewise, the C-terminal domain of the MCRV RdRp appears to play a similar role. In the MCRV RdRp, a loop and small α-helix from residues 1330–1339 form a relatively compact plug-like structure in this putative exit tunnel, resulting in a tunnel diameter of ~9–10 Å (Figs 5D and S11A). In the previously reported rotavirus VP1 structure, a short C-terminal α-helix occupies a similar position and extends ~15 Å into the (-)RiNA/dsRNA exit tunnel [14] though no plug is seen in λ3 [15] (S10 Fig). It should be noted however that two helices (residues 1283–1293, 1294–1304), also partially restrict the exit tunnel in MCRV RdRp. λ3 and rotavirus VP1 do not appear to contain analogous structures. Additionally, a transcription product exit tunnel is located along the RdRp surface nearest to the VP3 inner capsid layer (S11A Fig). This tunnel consists primarily of positively charged residues at the intersection of the C-terminal domain and the finger and palm subdomains. Interestingly, this tunnel is in line with a gap in the VP3 layer created by two VP3A and one VP3B.

### Interactions between the inner capsid and RdRp

While the N-termini of VP3B appears to be involved in the stabilization of the VP3 decamer and the inner capsid, the structural features also suggest that the N-termini of VP3A may play critical roles in “holding” the RdRp to the inner capsid; biochemical encapsidation assays also suggested a similar role for the inner capsid protein in rotavirus [39,40]. At the N-terminus of VP3A1, residues 59–69 form a loop that facilitates interactions with the RdRp (Figs 2C, 5E, 5F and 5G). The extended N-terminus of the VP3A2 interacts with the RdRp, with residues 40–48 forming an anti-parallel β-sheet with the residues 1220–1226 and 1390–1395 of the RdRp (Fig 5G). Together, these two unique conformations of N-termini in VP3A essentially bracket opposite sides of the RdRp and likely help anchor the polymerase to the capsid.

While the RdRp is located next to the 5-fold vertices, it is offset such that only three of the five VP3AB dimers contact the RdRp (Figs 2C and 5E). The interface is made up of primarily of the N-terminal and C-terminal RdRp domains and the apical domains of the three VP3As, though a portion of the carapace domain is involved in two of three VP3A subunits (VP3A1 and VP3A2). In the VP3B apical domain, residues 318–346 form a helix-loop-helix structure along the inner surface interacting with the residues 642–657 of the RdRp (Fig 5H). Of particular note, Lys642 and potentially His646 in the RdRp appear to form a salt bridge with Glu318 of VP3B (Fig 5H). Along with the aforementioned N-termini, these interactions likely help to anchor the RdRp in its offset position.

### Structure of tMCRV

In some members of *Reovirales*, capsids expand while transcribing [41], while others need to remove the outer capsid layer to activate transcription [42]. To better understand the structural changes of MCRV during transcription, we determined the structure of MCRV in a transcriptionally active state (tMCRV) with icosahedral symmetry and *D*_*5*_ symmetry imposed at 3.36 and 3.7 Å resolution, respectively.

Interestingly, at the current stated resolutions, we could not find any obvious conformational change for capsid proteins, including the inner capsid protein (VP3) and the outer capsid proteins (VP11 and VP12), nor was there any obvious change in capsid diameter. Furthermore, there were no gross structural differences in VP1 (the RdRp) despite the change in transcription state.

**Fig 6:**
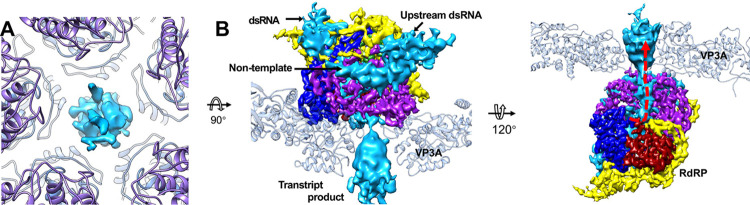
The extra densities in the transcribing MCRV (tMCRV). (A) The difference map between tMCRV and qMCRV shows the extra density (light blue) at the 5-fold vertex that has RdRp density underneath. The color schemes for models VP12 and VP3A are same as that of Fig 1B. The extra density is displayed at a contour level of about 8.3. (B) The extra density together with the RdRp of tMCRV is shown. Part of two copies of VP3A model is shown to indicate the position of the inner capsid. The extra density appears to be an RNA transcription product, together with the noncontinuous, non-template single strand RNA (also see S11 Fig). The RdRp density is displayed at a contour level of about 2.6. The color scheme for RdRp is same as that in Fig 5. In right panel, red dotted line indicates the transcript product exiting the RdRp and then through the gap among five VP3As at the 5-fold vertex.

Despite the lack of differences in the structural proteins, we did observe the RNA strands in the background of micrographs (S11C Fig) and extra densities in tMCRV map when compared to our qMCRV map. First, obvious extra densities were observed between the VP3 and VP12 subunits at the ten vertices (Fig 6A). After masking away capsid density, extra densities could be found in and around the RdRp of tMCRV (Figs 6B–6C and S11B). The density near the template entry site is consistent with double-strand RNA (dsRNA) with a diameter of about 30Å diameter, followed by a density of smaller diameter—likely a single RNA strand (Figs 6B and S11B)—inside the RdRp. It is reasonable to annotate this density inside the RdRp of tMCRV as the template strand. Continuing further from the template strand, the density becomes stronger and out of the RdRp and again has features of dsRNA. It could be annotated as the upstream dsRNA (Figs 6B, S11B and S11D–S11E). Opposite of the putative template strand and pointing to the transcript exit, the extra density becomes smaller in diameter again. This slim density travels along the surface of RdRp and through a gap between the VP3A subunits at the 5-fold vertex (Figs 6C, S11D and S11E). We annotated this density as the transcription product. Similar features have been observed in the transcribing CPV and [16,43], supporting the annotations of these densities and their relative roles in transcription. These features are similar to the reported elongation state of the CPV’s transcription [43], and may indicate that most particles we observed in the reconstruction could be in the transcription elongation state as well.

### Arrangement of the dsRNA genome

Like all the members in the *Reovirales*, the dsRNA genome of MCRV is highly organized and packaged inside the inner capsid around the RdRps (S12 Fig). In cross-sectional views, the *D*_5_ map clearly depicts at least six concentric genome layers organized around the ten RdRps with the outermost layer appearing to interact with the inner capsid. The gap between the adjacent layers is ~32 Å, similar to the 30 Å spacing previously reported in bluetongue virus [44] and cypovirus [16]. Strands in the outermost layers and closest to the RdRp are well defined, clearly showing major and minor grooves consistent with dsRNA; the diameter of the strands is ~23 Å with ~34 Å spacing between the grooves. The distance between the two adjacent strands in the same layer is about ~30 Å.

## Discussion

Our near-atomic resolution structure of MCRV has revealed the complete organization of the major capsid proteins, as well as ten RdRps, and the viral genome. While these structures certainly provide new insights into the capsid proteins and genomic interactions of dsRNA viruses, they also raise a number of fundamental questions.

### Reovirus origins

The relationship of MCRV to members of *Reovirales* has been difficult to resolve [2]. All extant members of *Reovirales* are divided into two clear families: the members of *Spinareoviridae*, having turrets and clamp proteins to hold together the inner shell; and the members of *Sedoreoviridae*, having internal capping enzymes and no turret or clamp. There has been perfect concordance between classification on these morphological features and the phylogenies of the polymerase sequence [21,45,46]; the polymerase is the only phylogenetically informative gene of members of the *Reovirales* over long timescales.

MCRV has been suggested to be classified within the non-turreted *Sedoreoviridae* as it lacks a capping turret [3] and because it expresses a protein with some homology to the internal capping enzyme of Seadornavirus [2]. Furthermore, MCRV VP1, VP3, and VP12 exhibit more structural similarity to rotavirus homologs than to any turreted reovirus. This agrees with the previous evidence placing MCRV in the non-turreted family [2,3]. However, we have now identified VP11 as a potential clamp protein (Fig 4), analogous to the cypovirus (CPV) LPP clamp [9], Fako virus clamp [21], orthoreovirus σ2 clamp [8], and aquareovirus VP6 clamp [35]. In the absence of detectable structural homology, it is unclear whether VP11 is a highly-divergent homolog of those clamp proteins or arose by convergent evolution due to specific constraints around stability. The large amount of divergence between the members of two families of *Reovirales* have made it unclear whether the turreted and non-turreted virus are sister clades or whether one is paraphyletic to the other. The presence of a clamp may have emerged after the divergence between Spinareoviridae and Sedoviridae.

### RdRp and its arrangement

Studies of RdRp positions inside the virion of reovirads have revealed some commonalities and also some enigmas. For both cypovirus and MRV, symmetry-mismatch reconstructions revealed 10 RdRps in a pseudo-*D*_3_ symmetric organization along with its 10 RNA segments; potentially each genome segment is specifically associated with one RdRp [16,17,19,47]. In GCRV and ARV, 11 RdRps along with their 11 RNA segments are arranged with a pseudo-*D*_3_ symmetric organization [13]. In Fako virus with 9 RNA segments, the RdRps are distributed at 10 locations with pseudo-*D*_3_ symmetry [20]. All the above-mentioned viruses belong to the *Spinareoviridae* family of *Reovirales*, which have turrets at their 5-fold vertices. Additionally, all their TECs are arranged with pseudo-*D*_3_ symmetry inside the capsid, though the locations of the unoccupied vertices within the capsid are different [47] (S1 Table). For *Sedoreoviridae*, although the high-resolution structures of Rotavirus and Bluetongue virus TECs have been determined [12,37], a defined arrangement of the TECs in the virus particles have not been reported. In rotavirus, it was reported that VP1 arrangement (the RdRp) has no symmetry and binds stochastically at one of the five possible positions at each of the 5-fold vertices [36]. MCRV, which has no turrets atop its 5-fold vertices, has 12 genome segments [2] but our results revealed 10 well-resolved RdRps. Additionally, these 10 RdRps are consistent with *D*_5_ symmetry, distinct from the pseudo-*D*_3_ symmetry found in Fako virus, cypovirus, MRV and ARV (S1 Table).

Only faint, blurred density can be found at the two pole vertices that do not have obvious RdRp density in either the *D*_5_- or *C*_1_-symmetry reconstructions. Our exhaustive pair-wise similarity analysis of the 60 views of the *C*_1_ map vertices related by icosahedral symmetry (S3 Fig) clearly demonstrated the *D*_5_ symmetry organization for the 10 vertices, each having clear density corresponding to a RdRp, while the remaining two pole vertices are distinct and have little density at a putative RdRp position. These results show that 10 well-resolved RdRp are arranged with *D*_5_ symmetry, not *D*_3_ as in other members of *Reovirales*. MCRV may have 11 or 12 vertex-associated RdRps, but if 12, the orientations of the polar RdRp are not correlated. This marked difference from all other characterized *Reovirales* members could be attributable to having 12 genome segments or to unique properties of this clade; distinguishing between these two hypotheses will require investigation of more species. Also, these techniques will only reveal capsid-associated RdRp; RdRp that do not have a consistent position/orientation with respect to the capsid require alternative approaches for detection [48].

In addition to the distinctive arrangement, the RdRp of MCRV has two conspicuous structural features: a specific protrusion on the innermost surface of the RdRp (Fig 5C), and a long loop that blocks the gap between the thumb and finger subdomains (Figs 5B and S10). In bluetongue virus, the RdRp has a “fingernail” motif that sits atop the finger subdomain and it pushes the terminal RNA to approach the template entry tunnel in a slightly different orientation [37]. The unique protrusion of MCRV RdRp is not linked to the finger subdomain, though the long loop is linked to the finger subdomain. The position of the protrusion is close to the template entry tunnel (Figs 5, S10 and S13), while the long loop points to different orientation; we hypothesize that the unique protrusion, despite not being directly linked to the finger domain, serves a similar function in MCRV as that of the fingernail domain in bluetongue virus.

### Multiple conformations and functions of VP3

As seen in the MCRV structure, the N-terminus of VP3 has several unique conformations and interacts with other VP3s, as well as the viral genome and the RdRp. In the VP3 dimer, the long “helix-loop-helix-loop” N-terminus of VP3B forms an interwoven network that stabilizes the shell (Fig 2B). The N-terminus in other dsRNA viruses with a pseudo *T* = 2 shell also have extend structures of the N-terminus to create a stable capsid shell (i.e. the N-anchor of capsid shell protein B (CSP-B) in cypovirus [9] and the N-terminus subdomain III of aquareovirus [28]). However, the long “helix-loop-helix-loop” interwoven structure observed in VP3B of MCRV is much more complex and, potentially, more stable.

The N-terminal fragments of three neighboring VP3As around the five-fold axes interact with the genome (Fig 2C); N-terminal fragments have been shown to be involved in RNA organization and movement around the RdRp [49,50]. In bluetongue virus, evidence suggests that the smallest genome segment triggers RNA–RNA interaction and further recruits the medium to larger size ssRNA genome segments to be packaged into the capsid, with the inner capsid protein playing some correcting roles in the whole process [51]. This result gives clues that the packaging and organizations of genome segments together with the inner capsid may direct the RdRps organization.

It has been widely reported that the inner capsid proteins of *Reovirales* members, especially the N-terminal region, are involved in the regulation of the polymerase activity, genome replication, and mRNA transcription [12,49,52,53,54]. In cypovirus, the N-terminus of the inner capsid protein also interacts with the RdRp; the N-terminal helices of two CSPs interact with the RdRp to affect its activities [17]. In bluetongue virus, the N-termini of five VP3As have five slightly different conformations that interact with the RdRp. Similarly, N-terminal fragments from two VP3As (VP3A1, VP3A2) interact with the RdRp (Fig 5F and 5G). In VP3A2, not only does the α-helix (residues 51–76) insert into the RdRp in a manner similar to that of cypovirus, but residues 40–48 form a strand and interact with two strands of the bracelet domain of the RdRp to form an anti-parallel β-sheet (Fig 5G). Forming one strand of this β-sheet, residues 1220–1204 of the RdRp directly link to “module A” of the bracelet domain, as described in cypovirus. In the transcription and quiescent stages, module A and this region of the capsid protein undergo conformational changes [17]. Secondly, in the same VP3A, apical domain residues 309–348 are proximal to residues 1350–1364 of RdRp, which directly link to the “α-helix plug”. Therefore, it is justifiable to surmise that the aforementioned apical domain fragment can regulate the “plug” as its conformation is changed. Additional studies have shown that not only can the N-terminus of the inner capsid protein be involved in the regulation, but also that the residues at or beyond the apical domain are critical for the RdRp activities [52]. Our results provide clear structural evidence that the residues at the apical domain might be critical to regulate the “plug” of RdRp.

The N-terminal fragment of one VP3B is found to interact with the finger subdomain of the RdRp (Fig 5F and 5H). In cypovirus, this area is the interface between the NTPase of VP4 and the finger subdomain [17]. In MCRV, no obvious extra density can be found here. However, the interface is close to the entries of two RdRp tunnels: the NTP entry and the template entry tunnels. Previous studies have demonstrated that VP2 (the inner capsid shell protein) in rotavirus can strongly bind to the mRNA template during genome replication [54]. Further studies have shown more structural evidence that the N-terminal regions of the inner capsid shell protein homologs serve as transcriptional regulating factors for RdRp [12].

In summary, our results reveal several unique features of the unusual, 12-segmented MCRV. Despite its classification into the turrret-less/clamp-less *Sedoreoviridae*, MCRV does contain “clamp”-like proteins (VP11) in its outer capsid. These clamp proteins appear to be crucial in maintaining capsid stability. Additionally, the N-termini of the inner capsid proteins, VP3, have multiple conformations with vastly different functions. The N-terminus of VP3B has a unique “helix-loop-helix-loop” motif, suggesting it may play roles in capsid assembly and stable, while the N-termini of VP3A may help to anchor the RdRp in its offset location on the inner surface of the capsid. The RdRps are positioned off the 5-fold axes, and their arrangement in the capsid obeys *D*_5_ symmetry. Ten RdRp arrange in a correlated, *D*_5_ configuration within the capsid, while the remaining two fivefold vertices could bind zero, one, or two RdRps. Further differentiating MCRV from sister viruses, the RdRp has two unique features: a protrusion of the N-domain and long loop on the top of the finger domain which may play a role in transcription. The extra densities in tMCRV RdRps demonstrate its transcriptional ability. Together these findings help to establish a more complete understanding of the organization of the members of *Reovirales*, the structure and function for individual proteins, and the evolutionary relationships among *Reovirales* members.

## Methods

### Virus isolation and purification

The mud crab *Scylla serrata* was infected by MCRV. Virus purification was performed according to the previous method [4] with slight modification as described below. The gills of the mud crab infected by MCRV were homogenized in PBS (2.7mM KCl, 137mM NaCl, 10mM Na_2_HPO_4_, 2mM KH_2_PO_4_, pH 7.4) at 4°C. The homogenized samples were centrifuged for 1h at 10,000g to discard the tissue and cell debris. The supernatant was then centrifuged at 200,000g for 2 hours. The pellet was re-suspended in PBS buffer and then loaded on a 15~45% (w/w) CsCl gradient and centrifuged at 200,000g for 8 hours. The recovered fractions were diluted in PBS buffer and centrifuged for 2 hours at 200,000g. The pellet was re-suspended in PBS buffer and was checked by negative stain TEM (JEM 2010) to make sure the concentration and purity was adequate for further cryo-EM imaging. For negative stain EM, 3 μl of sample was applied to a glow-discharged carbon-film coated grid for 1 min. After removal of excess buffer, the grid was stained with 3% phosphotungstic acid (PTA) (w/v) for 1 min. The excess PTA was then removed by filter paper and the grid was dried in the air.

### Cell-free transcription reaction

For the transcription reaction, purified MCRV particles were incubated in transcription buffer (70 mM Tris–HCl, pH 8, 4 mM rATP; 2 mM rGTP; 2 mM rCTP and 2 mM rUTP; 1 mM S-adenosylmethionine; 1 mM recombinant Rnase Inhibitor; 10 mM MgAc2; 10 mM NaAc) for 10 min at 31°C prior to plunge-freezing for cryo-EM [41,55]. RNA transcription was confirmed with a control experiment using [α-32P] UTP in the transcription reaction mixture before the sample was used for cryo-EM.

### Cryo-EM sample grid preparation and data collection

The R1.2/1.3 copper Quantifoil holey grids were coated a fresh thin layer of continuous carbon film just before cryo-EM sample freezing. 2.5 μl MCRV sample was applied to the grids, blotted, and then flash-frozen in precooled liquid ethane using a FEI Vitrobot Mark IV at 100% humidity. The frozen grids were stored in liquid nitrogen for subsequent cryo-EM data acquisition.

The frozen grids were loaded into an FEI Titan Krios electron microscope operated at 300 kV. Cryo-EM micrographs were recorded on an FEI Falcon II direct electron detection camera at 75000 × nominal magnification with the calibrated pixel size of 1.09 Å at the sample level. The intended defocuses ranged from 0.6 to 3 μm. The dose rate on the sample was 23 eÅ^-2^s^-1^. The data were recorded as movies of 16 frames with total dose of 25 eÅ^-2^ and exposure time of 1.1 s. 3595 were used for final image processing and 3D reconstruction for the qMCRV; while for tMCRV, 2323 movie were used for final reconsctruction.

### Image processing and 3D reconstruction

The movies were aligned and drift-corrected using the GPU-accelerated program *Motioncorr* V2.0 [56]. The *EMAN2* program *e2boxer*.*py* [57] was used to select 58,095 particles from 3,595 micrographs (Fig 1A) and 2,1945 particles from 2,323 movies for qMCRV and tMCRV, respectively. Some of the selected particles were “empty” without encpasidated genome. It is not known whether these particles were assembled without RNA or if they lost RNA after capsid assembly (either before or after second-strand synthesis). These empty particles were manually separated from other genome-containing particles for further data analysis. The particles were extracted directly from the movie frames with dose/radiation damage dependent weighting using the *batchboxer*.*py* program in *JSPR* [58]. Contrast transfer function parameters were determined automatically using *fitctf2*.*py* and then visually validated using EMAN *ctfit* program. The dataset was divided into two halves and all subsequent image processing, including construction of initial *de novo* models and iterative refinements, was performed on each of the two subsets independently using the *JSPR* software. All resolutions were assessed with “gold standard” Fourier Shell Correlation using 0.143 criterion [59,60] (S1 Fig). The particles were first reconstructed as icosahedral particles as described previously [58]. Briefly, the initial models were built by iteratively refining randomly assigned initial orientations using 4x binned particles. The particle parameters were then transferred to 2× binned particles and particles without binning for high resolution refinements. The icosahedral reconstructions were then used as the starting point to perform *C*_1_ asymmetric reconstructions of the MCRV particles using the symmetry relaxation method that we previously developed [24,25]. Without imposing any symmetry, the 3D reconstructions quickly converged to reveal well-resolved RdRp protein densities under the 5-fold vertices (S2 Fig). By visual inspection and quantitative evaluation of the similarities of the RdRp densities and their relative orientations (S2 and S3 Figs), we found RdRp densities at 10 of the 12 vertices that were well resolved and related by *D*_5_ symmetry, while the remaining 2 vertices had only weak RdRp densities. To further improve the RdRp densities, we imposed *D*_5_ symmetry in subsequent reconstructions (S2 and S3 Figs).

### Symmetry assessment: decoy method

Particle position, scale, defocus, and astigmatism were fixed throughout refinement and the orientation was allowed to vary only among icosahedrally-equivalent Euler angles.

A RdRp was first segmented from the *D*_5_-symmetrized map with UCSF Chimera. Decoys were then constructed by summing RdRp and capsid maps together. The icosahedrally-symmetrized capsid map was masked to remove density at each of the 60 possible RdRp sites. The intensity of the RdRp and capsid were matched by multiplying the RdRp map by a constant. Next, a number of RdRp maps were added to the capsid at specified icosahedrally-related locations. This decoy map was then filtered to 8Å resolution. Various decoys were generated with RdRp placements as follows: random placements of 8 RdRps (subject to the constraint of only one RdRp per five-fold axis), a CPV-like configuration of 10 RdRps, a *D*_5_ configuration of 10 RdRps, a *D*_5_ configuration plus one or two polar RdRps in each possible configuration, and a *D*_5_ configuration of 10 RdRps, where each RdRp is turned 144° compared to the structure reported herein.

Particles were subjected to iterative multi-reference alignment and reconstruction starting with the decoys as models. Per-particle correlations to the various decoys were evaluated to assess which decoy best reflects the particle population.

### Model building and assessment

Models for the capsid proteins and RdRp were constructed using *de novo* methods as previously described [61,62]. Briefly, UCSF *Chimera* [63] was used to manually segment out individual capsid proteins based on visual inspection. Once segmented, individual subunits were aligned using a combination of the *e2foldhunter*.*py* program [64,65] from *EMAN2* and the “Fit in map” function in Chimera. From this alignment, average maps for each of RdRp, VP3A, VP3B, VP12 and VP11 were constructed. Initial backbone models were constructed directly from the density map with Pathwalking (*e2pathwalker*.*py* in EMAN2) [64]; only a density threshold and the corresponding number of residues in a subunit were provided as additional inputs. Pathwalking produces a non-directional Cα backbone trace and visual inspections of the models in the corresponding maps were used to determine directionality. The primary sequence of the corresponding protein was then threaded onto the model and converted to an all-atom model with Remo [66]. Iterative real-space refinement and visual optimization were done using *Phenix* [67] and *Coot* [68], respectively. Model quality was monitored by examining fit-to-density, Ramachandran outliers, clashes and rotamers. For models from the asymmetric map, individual subunit models were first fit to the corresponding regions of the map and the refined with Phenix real space refinement. For regions with weak density, extra density or where there were large structural rearrangements, manual model building was done with *Coot*, followed by additional real-space refinement steps. Once models for a complete asymmetric unit were refined, five neighboring asymmetric unit models were placed in adjacent asymmetric unit positions of the density map in order to capture all possible interactions. A final round of *Phenix* real-space refinement was used to minimize clashes between subunits in neighboring asymmetric units. The final asymmetric units were then transformed to construct a complete capsid model. Final models were assessed using *molprobity* [69] for assessing model quality (S2 Table). Figures and model analysis were done in *UCSF Chimera* and *Coot*; subunit interactions were examined using PDBe *Pisa* [70].

## Supporting information

S1 Table Comparison of reoviruses with different numbers of genome segments/RdRps. (DOCX)

S2 Table Model quality statistics. (DOCX)

S1 Script Python script symAngleDifferences.py that was used to compute the count numbers in S1C Fig. (PY)

S1 Movie The icosahedral structure of MCRV. (MOV)

S1 Fig Cryo-EM of MCRV. (A) A representative cryo-EM image of MCRV particles embedded in vitreous ice. (B) The Fourier shell correlation curves of the icosahedral and D_5_ reconstructions of MCRV and tMCRV. Resolutions are measured at 3.1 Å and 3.4 Å for MCRV with icosahedral and D_5_ symmetry, respectively, and 3.36 Å and 3.7 Å for tMCRV with icosahedral and D_5_ symmetry, respectively. Resolution measurements were based on the “gold-standard” FSC = 0.143 criterion. (C) Cartoon view of the MCRV. The polymerase (VP1), inner capsid proteins (VP3A and VP3B), clamp protein (VP11) and outer capsid protein (VP12) are colored in red, cornflower blue, orange, light green and medium purple, respectively.
(TIF)

S2 Fig Convergence process of *ab initio* asymmetry reconstruction of MCRV. XC row: central section perpendicular to icosahedral 2-fold axis (i.e. X axis). Z1 to Z4 rows: sections perpendicular to icosahedral 5-fold axis (i.e. Z axis) at locations indicated by the dashed lines in XC row. The icosahedral reconstruction was used as starting model to initiate the iterative symmetry relaxation alignment and asymmetric reconstruction process until convergence. The XC and Z sections of the map for each iteration are shown in a column indicated by the iteration numbers (0 to 8). The black arrows indicate the RdRp densities are clear resolved by iteration 4.
(TIF)

S3 Fig Discovery of the D_5_ symmetry arrangement of MCRV’s RdRps. (A) MDS clustering of the C1 reconstruction vertices in all 60 icosahedral related views. The C1 map was rotated to all 60 icosahedral related views and similarities among all possible combinations of the vertices were computed. The 6 apparent clusters were labeled 1 to 6. Each cluster consisted of 10 views. (B) Analysis of the distribution of the angles between all pairs (10*9/2 = 45) among the 10 views in each of the 6 clusters. (C) List of the expected distribution of angles between all pairs of symmetry operations for the listed symmetries. All the distributions in (B) matched that of *D*_*5*_ symmetry (bold) suggesting that the RdRps located at the 10 vertices were arranged in *D*_*5*_ symmetry, a subset of icosahedral symmetry. (D) Central section view of C1 (left) and *D*_*5*_ (right) symmetry reconstructions. The middle column (C1—*D*_*5*_ view) shows the same C1 map (left) but re-oriented in the same *D*_*5*_ view as that in the right column (*D*_*5*_ symmetry map) then imposed with *D*_*5*_ symmetry. XC: central section perpendicular to icosahedral 2-fold axis (i.e. X axis). Z1 to Z4: sections perpendicular to icosahedral 5-fold axis (i.e. Z axis) at locations indicated by the dashed lines in XC row. The numbers in table (C) were calculated using the python script symAngleDifferences.py (S1 Script).
(TIF)

S4 Fig The arrangement of RdRps inside empty MCRV. (A) 3D classification of empty MCRV reveals 6 classes with 10 well resolved RdRp densities arranged with *D*_5_ symmetry, after masking away the capsid density and symmetry expansion. The densities at the remaining two vertices are substantially weaker. The dot lines show the potential axes although we didn’t impose the symmetry during the refinement. (B)10 well resolved RdRp densities can be seen in the reconstruction from empty particles. An icosahedron is displayed in orange as a visual guide, and two icosahedral vertices contain weaker densities. Numbers 1–10 mark the 10 RdRps. (C) Thirteen decoys were constructed by placing 5 closest neighboring RdRps, (such as, if we select RdRp1 marked in (B), then the other closest RdRps will include RdRp2, 5, 6 and 10) with random orientation. Decoy 13 is in agreement with that of *D*_5_ symmetry. For each of the thirteen, the fraction of particles aligning to the corresponding decoy is shown in gray bar. The RdRps of each decoy are displayed and linked to the corresponding bar by dot lines. One of the thirteen random decoys reflected the underlying data better than did the other decoys. The results demonstrates that empty MCRV particles matched best with *D*_5_ symmetry.
(TIF)

S5 Fig The structure of VP3. (A) Density map (gray) of VP3A superimposed with its atomic model (cornflower blue). (B) Zoom-in regions of the density map (black net) superimposed with atomic model, demonstrating the quality of cryo-EM map VP3A and that of the refined atomic model.
(TIF)

S6 Fig Comparison between VP3A and VP3B. The atomic models for VP3A (cornflower blue) and VP3B (orange) are shown overlaid. There is considerable difference between the VP3A and VP3B subunits. Specifically, the biggest differences are at the distal edge of the dimerization domain (red circle), in the carapace domain (green circle) and on the inner surface of the carapace domain (black circle), and the extended N-terminus of VP3B.
(TIF)

S7 Fig The structure of VP12. (A) Density map (gray) of VP12 superimposed with its atomic model (medium purple). (B) Zoom-in regions of the density map (black nets) superimposed with atomic model (medium purple), demonstrating the quality of cryo-EM map VP12 and that of the refined atomic model.
(TIF)

S8 Fig The structure of VP11. (A) Density map (gray) of VP11 superimposed with its atomic model (green). Right panel shows that zoom-in regions of the density map superimposed with atomic model, demonstrating the quality of cryo-EM map VP11 and that of the refined atomic model. (B) Alignment of VP11A and VP11B (cyan) reveals few conformation differences between them. The main differences are at the termini (blue arrows) and two peripheral loops (black arrows). (C) The sequence and secondary structural elements are indicated. The color schemes for secondary structure are the same as the model in Fig 4D.
(TIF)

S9 Fig Interactions between the VP11A and its neighbor proteins. (A) VP11A interacts with the surrounding VP12s. (B) The hydrogen bonds between the VP11A and two VP12 proteins. (C) The interacting amino acids at the interface between VP11 and VP12 are indicated. (D) VP11A also interacts with the VP3s. (E) The hydrogen bond between the VP11A and VP3A. (F) The interacting amino acids at the interface between VP11A and VP3 are indicated. (G) VP11B clamps the two neighbor VP3 pentamers together. (H) The hydrogen bond between the VP11B and VP3B. (I) The interacting amino acids at the interface between VP11B and VP3 are listed. (J) The hydrophobic interactions between VP11B and VP3B, made by Phe130 in VP11 and Val106, Val 821, Leu822 in VP3. The model of VP11B is colored with cyan, while the model of VP3B is colored with orange.
(TIF)

S10 Fig A Comparison of RdRps of several *Reovirales* family members. The color scheme is identical to that in Fig 5. The structure inside the black circle is the unique protrusion of the N-domain in MCRV. Black arrows indicate the priming loop; red arrows indicate the C-terminal plug. The orange arrow indicates the unique long loop blocking the gap between the thumb and finger subdomain of MCRV RdRp. The unique “fingernail motif” of bluetongue virus is colored in cyan.
(TIF)

S11 Fig The tunnels of RdRp and major differences in RdRp between MCRV and tMCRV. (A) indicates the four tunnels of RdRp. The asterisks indicate the positions of tunnels. The color schemes of RdRp and capsid are same as that in Figs 1 and 5. (B) indicates the RdRp map together with the extra densities (colored with light blue) in tMCRV. The orientation of each image view, together with the color schemes of RdRp and capsid are the same as that in A. (C) shows a typical cryo-EM image of tMCRV particles. Black arrows show the RNA strands. (D) The difference map (light blue) shows the extra density in the center of the RdRp. The color scheme of the RdRp model is same as that of Fig 5. The inner capsid position is shown by partial models of VP3A. (E), The extra density has features concordant with downstream dsRNA, the ssRNA template, the upstream dsRNA together with the noncontinuous non-template single strand RNA, and the transcript product. The density of non-template ssRNA is not continuous and is indicated by dot lines. The difference map is shown at a contour level of roughly 8.3.
(TIF)

S12 Fig The organization of the RdRps and dsRNA genome segments of MCRV. Density maps without the two capsid shells viewed along the 5-fold axis reveal that there are two kinds of icosahedral 5-fold vertices (A, B). Two of the twelve vertices have less density around the 5-fold axes (A), while at the remaining 10 vertices there is obvious RdRp density (orange) slightly offset from the 5-fold axes (B). Panels (C) and (D) show the maps along the 3- and 2-fold axis, respectively. 5-, 3-, and 2-fold axes are marked in pentagon, triangle and ellipse respectively. The map, except for the densities of polymerase, is radially colored.
(TIF)

S13 Fig Comparison of the RdRps of MCRV and Bluetongue virus. The color scheme is identical to that in S9 Fig. The left column and middle column are the polymerase of Bluetongue and MCRV respectively. The right column displays the alignment of tow polymerases. The unique protrusion (yellow) of MCRV’s N-terminal domain is displayed. The alignment of the two RdRps reveals that the unique protrusion’s position is close to that of the fingerail motif of bluetongue virus RdRp.
(TIF)
