# Supplementary material for: The structure of a 12-segmented dsRNA reovirus: New insights into capsid stabilization and organization
Source: PLoS Pathog. 2023 Apr 21;19(4):e1011341. doi: 10.1371/journal.ppat.1011341 (PMC10155992; doi:10.1371/journal.ppat.1011341)
Supplement: S1 Table — (DOCX) [file ppat.1011341.s001.docx]

**S1 Table comparison table for reoviruses with different numbers of genome/RNA dependent RNA-Polymerase (RdRP)**

| Virus | Genus | Genome segments | vertices occupied by RdRPs | RdRP symmetry | Turreted | Protein layers | references |
| --- | --- | --- | --- | --- | --- | --- | --- |
| Fako virus (FAKV) | *Dinovernavirus* | 9 | 10 | D2  (pseudo D3) | ✔ | 1 | [1] |
| cytoplasmic polyhedrosis virus (CPV) | *Cypovirus* | 10 | 10 | D3 | ✔ | 1 | [2, 3] |
| mmmalian reovirus  (MRV) | *Orthoreovirus* | 10 | 10 | D3 | ✔ | 2 | [4] |
| bluetongue virus | *Orbivirus* | 10 | ? | ? | ✖ | 3 | [5] |
| grass carp reovirus (GCRV)(ARV) | *Aquareovirus* | 11 | 11 | D3 | ✔ | 2 | [6, 7] |
| rotavirus (RRV) | *Rotavirus* | 11 | ? | no | ✖ | 3 | [8] |
| Mud crab reovirus (MCRV) | *?* | 12 | 10+ | D5 | ✖ | 2 | this paper |

**References:**

1. Kaelber JT, Jiang W, Weaver SC, Auguste AJ, Chiu W. Arrangement of the Polymerase Complexes inside a Nine-Segmented dsRNA Virus. Structure. 2020;28(6):604-12 e3. doi: 10.1016/j.str.2020.01.011. PubMed PMID: 32049031; PubMed Central PMCID: PMCPMC7289189.

2. Liu H, Cheng L. Cryo-EM shows the polymerase structures and a nonspooled genome within a dsRNA virus. Science. 2015;349(6254):1347-50. Epub 2015/09/19. doi: 10.1126/science.aaa4938. PubMed PMID: 26383954.

3. Zhang X, Ding K, Yu X, Chang W, Sun J, Hong Zhou Z. In situ structures of the segmented genome and RNA polymerase complex inside a dsRNA virus. Nature. 2015. Epub 2015/10/28. doi: 10.1038/nature15767. PubMed PMID: 26503045.

4. Pan M, Alvarez-Cabrera AL, Kang JS, Wang L, Fan C, Zhou ZH. Asymmetric reconstruction of mammalian reovirus reveals interactions among RNA, transcriptional factor micro2 and capsid proteins. Nat Commun. 2021;12(1):4176. Epub 20210707. doi: 10.1038/s41467-021-24455-4. PubMed PMID: 34234134; PubMed Central PMCID: PMCPMC8263624.

5. He Y, Shivakoti S, Ding K, Cui YX, Roy P, Zhou ZH. In situ structures of RNA-dependent RNA polymerase inside bluetongue virus before and after uncoating. Proceedings of the National Academy of Sciences of the United States of America. 2019;116(33):16535-40. doi: 10.1073/pnas.1905849116. PubMed PMID: WOS:000481404300060.

6. Ding K, Nguyen L, Zhou ZH. In Situ Structures of the Polymerase Complex and RNA Genome Show How Aquareovirus Transcription Machineries Respond to Uncoating. Journal of virology. 2018;92(21). doi: ARTN e00774-1810.1128/JVI.00774-18. PubMed PMID: WOS:000447139100006.

7. Wang XR, Zhang FX, Su R, Li XW, Chen WY, Chen QX, et al. Structure of RNA polymerase complex and genome within a dsRNA virus provides insights into the mechanisms of transcription and assembly. Proceedings of the National Academy of Sciences of the United States of America. 2018;115(28):7344-9. doi: 10.1073/pnas.1803885115. PubMed PMID: WOS:000438050900070.

8. Jenni S, Salgado EN, Herrmann T, Li ZL, Grant T, Grigorieff N, et al. In situ Structure of Rotavirus VP1 RNA-Dependent RNA Polymerase. Journal of molecular biology. 2019;431(17):3124-38. doi: 10.1016/j.jmb.2019.06.016. PubMed PMID: WOS:000482872100008.
