# Supplementary material for: The structure of a 12-segmented dsRNA reovirus: New insights into capsid stabilization and organization
Source: PLoS Pathog. 2023 Apr 21;19(4):e1011341. doi: 10.1371/journal.ppat.1011341 (PMC10155992; doi:10.1371/journal.ppat.1011341)
Supplement: S2 Table — (DOCX) [file ppat.1011341.s002.docx]

**S2 Table Model quality analysis**

|  | MCRV (Icos) | MCRV (D5) |
| --- | --- | --- |
| Ramachandran outliers | 00% | 0.23% |
| Ramachandran favored | 96.23% | 91.37% |
| Rotamer outliers | 0.57% | 0.58% |
| C-Beta deviations | 0 | 5 |
| Clashscore | 4.92 | 10.38 |
| RMS (bonds) | 0.007 | 0.006 |
| RMS (angles) | 1.133 | 1.091 |
| MolProbity score | 1.51 | 2.04 |
| CC_model vs map (masked) | 0.85 | 0.79 |
