# Supplementary figures and images for: The structure of a 12-segmented dsRNA reovirus: New insights into capsid stabilization and organization
Source: PLoS Pathog. 2023 Apr 21;19(4):e1011341. doi: 10.1371/journal.ppat.1011341 (PMC10155992; doi:10.1371/journal.ppat.1011341)

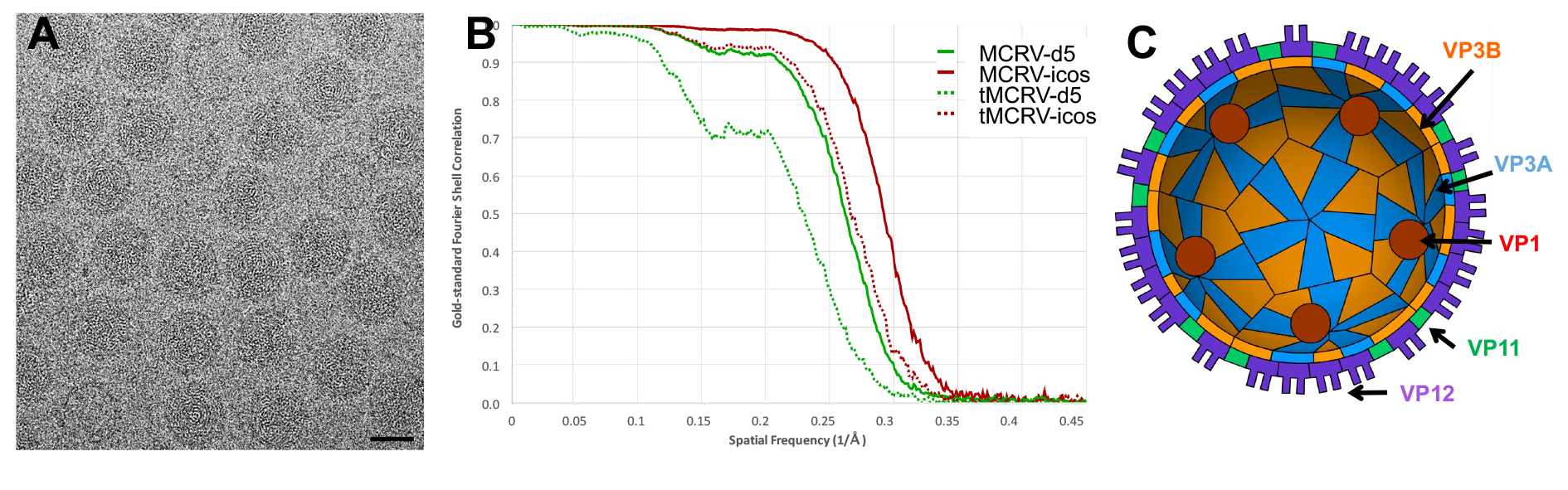

Supplement: S1 Fig — (A) A representative cryo-EM image of MCRV particles embedded in vitreous ice. (B) The Fourier shell correlation curves of the icosahedral and D5 reconstructions of MCRV and tMCRV. Resolutions are measured at 3.1 Å and 3.4 Å for MCRV with icosahedral and D5 symmetry, respectively, and 3.36 Å and 3.7 Å for tMCRV with icosahedral and D5 symmetry, respectively. Resolution measurements were based on the “gold-standard” FSC = 0.143 criterion. (C) Cartoon view of the MCRV. The polymerase (VP1), inner capsid proteins (VP3A and VP3B), clamp protein (VP11) and outer capsid protein (VP12) are colored in red, cornflower blue, orange, light green and medium purple, respectively. (TIF) [file ppat.1011341.s005.tif]

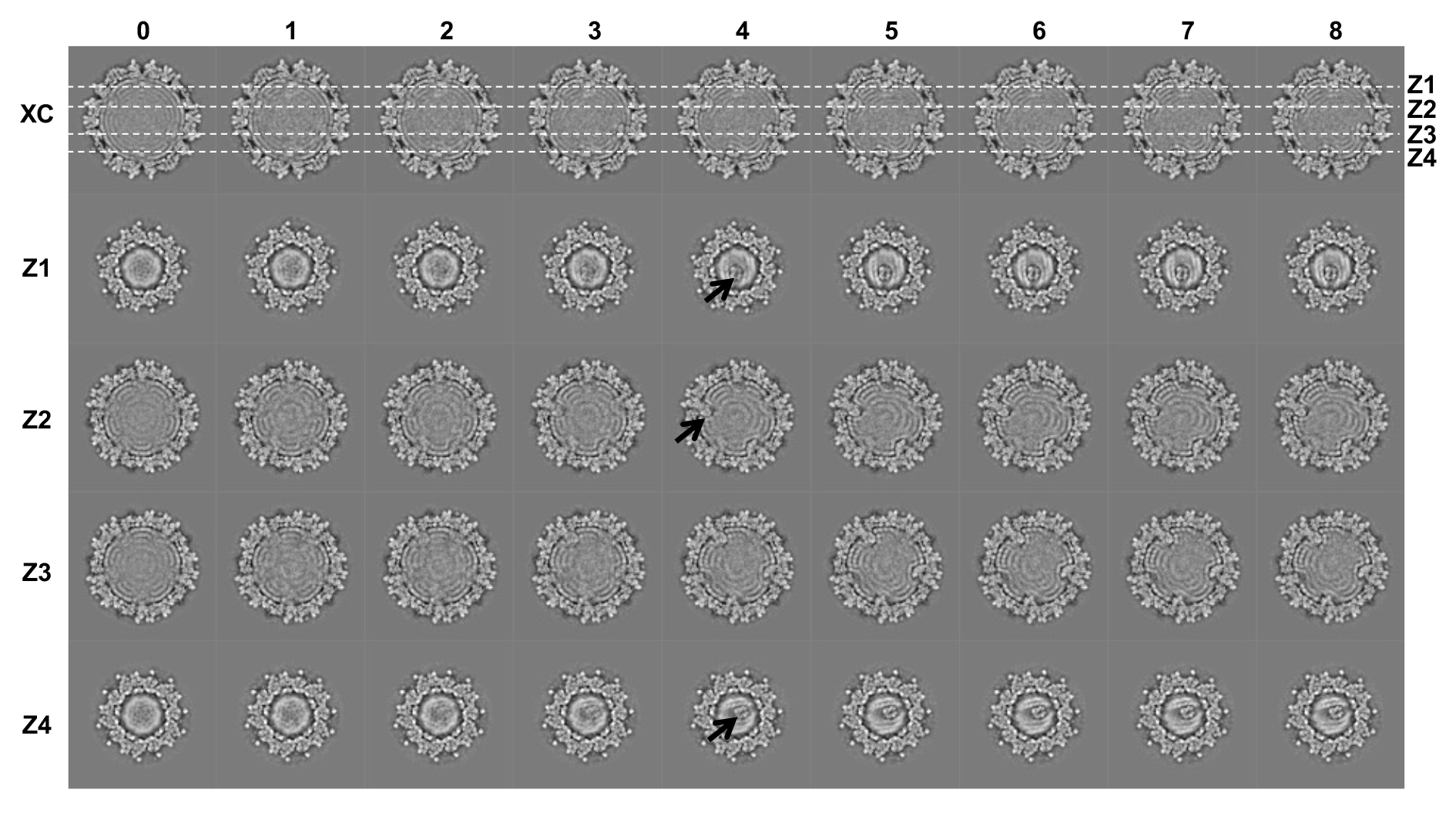

Supplement: S2 Fig — XC row: central section perpendicular to icosahedral 2-fold axis (i.e. X axis). Z1 to Z4 rows: sections perpendicular to icosahedral 5-fold axis (i.e. Z axis) at locations indicated by the dashed lines in XC row. The icosahedral reconstruction was used as starting model to initiate the iterative symmetry relaxation alignment and asymmetric reconstruction process until convergence. The XC and Z sections of the map for each iteration are shown in a column indicated by the iteration numbers (0 to 8). The black arrows indicate the RdRp densities are clear resolved by iteration 4. (TIF) [file ppat.1011341.s006.tif]

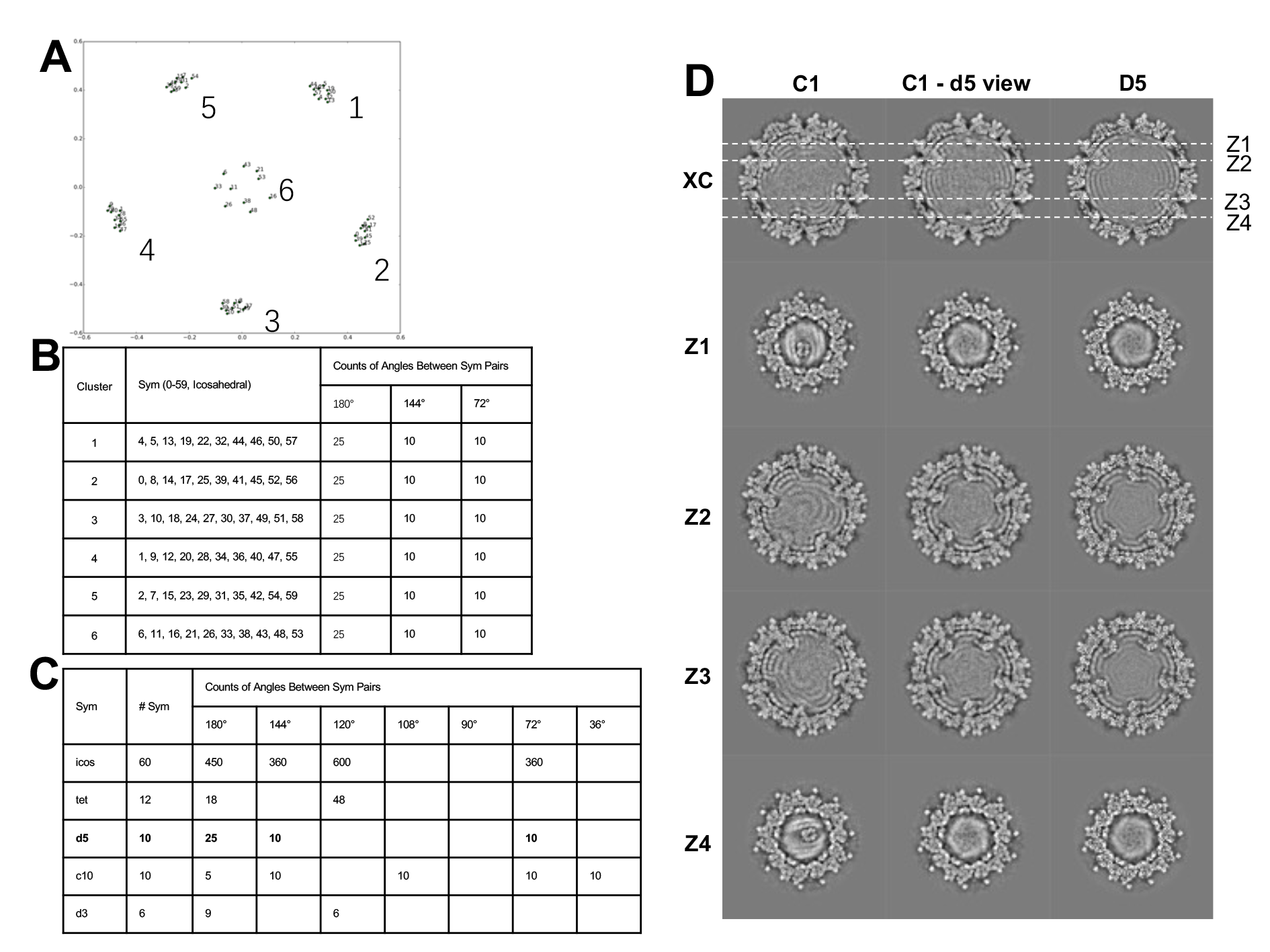

Supplement: S3 Fig — (A) MDS clustering of the C1 reconstruction vertices in all 60 icosahedral related views. The C1 map was rotated to all 60 icosahedral related views and similarities among all possible combinations of the vertices were computed. The 6 apparent clusters were labeled 1 to 6. Each cluster consisted of 10 views. (B) Analysis of the distribution of the angles between all pairs (10*9/2 = 45) among the 10 views in each of the 6 clusters. (C) List of the expected distribution of angles between all pairs of symmetry operations for the listed symmetries. All the distributions in (B) matched that of D5 symmetry (bold) suggesting that the RdRps located at the 10 vertices were arranged in D5 symmetry, a subset of icosahedral symmetry. (D) Central section view of C1 (left) and D5 (right) symmetry reconstructions. The middle column (C1—D5 view) shows the same C1 map (left) but re-oriented in the same D5 view as that in the right column (D5 symmetry map) then imposed with D5 symmetry. XC: central section perpendicular to icosahedral 2-fold axis (i.e. X axis). Z1 to Z4: sections perpendicular to icosahedral 5-fold axis (i.e. Z axis) at locations indicated by the dashed lines in XC row. The numbers in table (C) were calculated using the python script symAngleDifferences.py (S1 Script). (TIF) [file ppat.1011341.s007.tif]

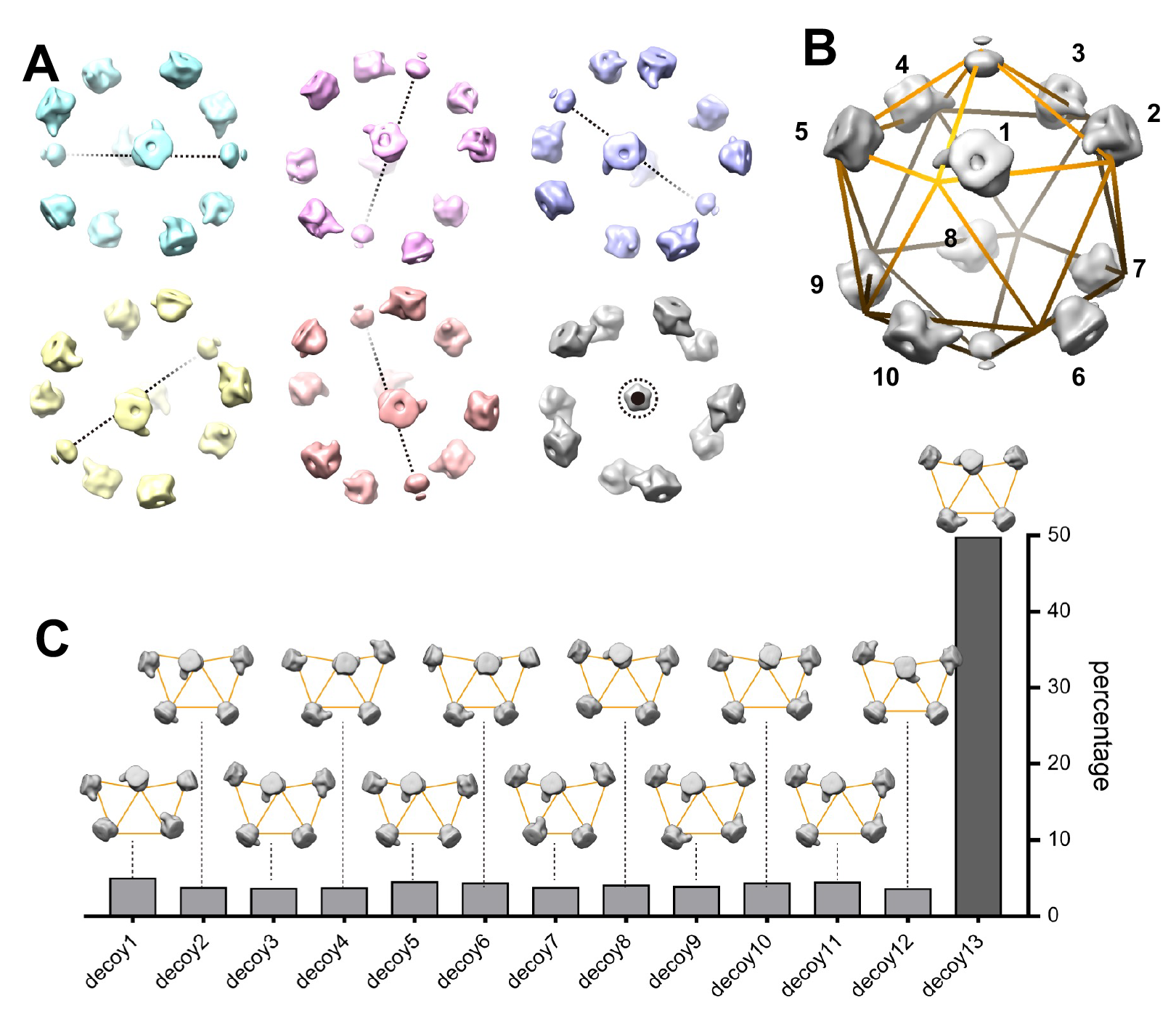

Supplement: S4 Fig — (A) 3D classification of empty MCRV reveals 6 classes with 10 well resolved RdRp densities arranged with D5 symmetry, after masking away the capsid density and symmetry expansion. The densities at the remaining two vertices are substantially weaker. The dot lines show the potential axes although we didn’t impose the symmetry during the refinement. (B)10 well resolved RdRp densities can be seen in the reconstruction from empty particles. An icosahedron is displayed in orange as a visual guide, and two icosahedral vertices contain weaker densities. Numbers 1–10 mark the 10 RdRps. (C) Thirteen decoys were constructed by placing 5 closest neighboring RdRps, (such as, if we select RdRp1 marked in (B), then the other closest RdRps will include RdRp2, 5, 6 and 10) with random orientation. Decoy 13 is in agreement with that of D5 symmetry. For each of the thirteen, the fraction of particles aligning to the corresponding decoy is shown in gray bar. The RdRps of each decoy are displayed and linked to the corresponding bar by dot lines. One of the thirteen random decoys reflected the underlying data better than did the other decoys. The results demonstrates that empty MCRV particles matched best with D5 symmetry. (TIF) [file ppat.1011341.s008.tif]

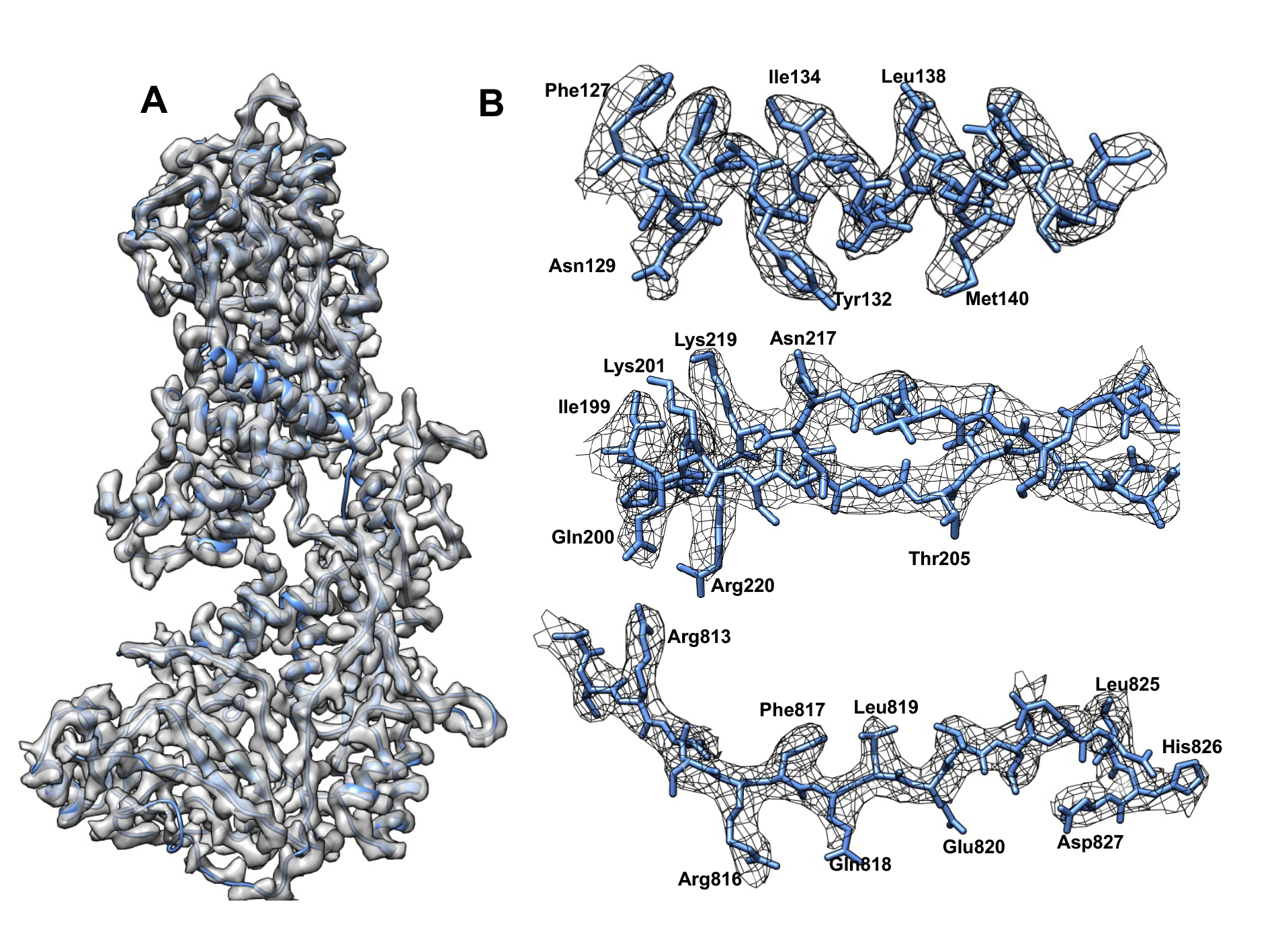

Supplement: S5 Fig — (A) Density map (gray) of VP3A superimposed with its atomic model (cornflower blue). (B) Zoom-in regions of the density map (black net) superimposed with atomic model, demonstrating the quality of cryo-EM map VP3A and that of the refined atomic model. (TIF) [file ppat.1011341.s009.tif]

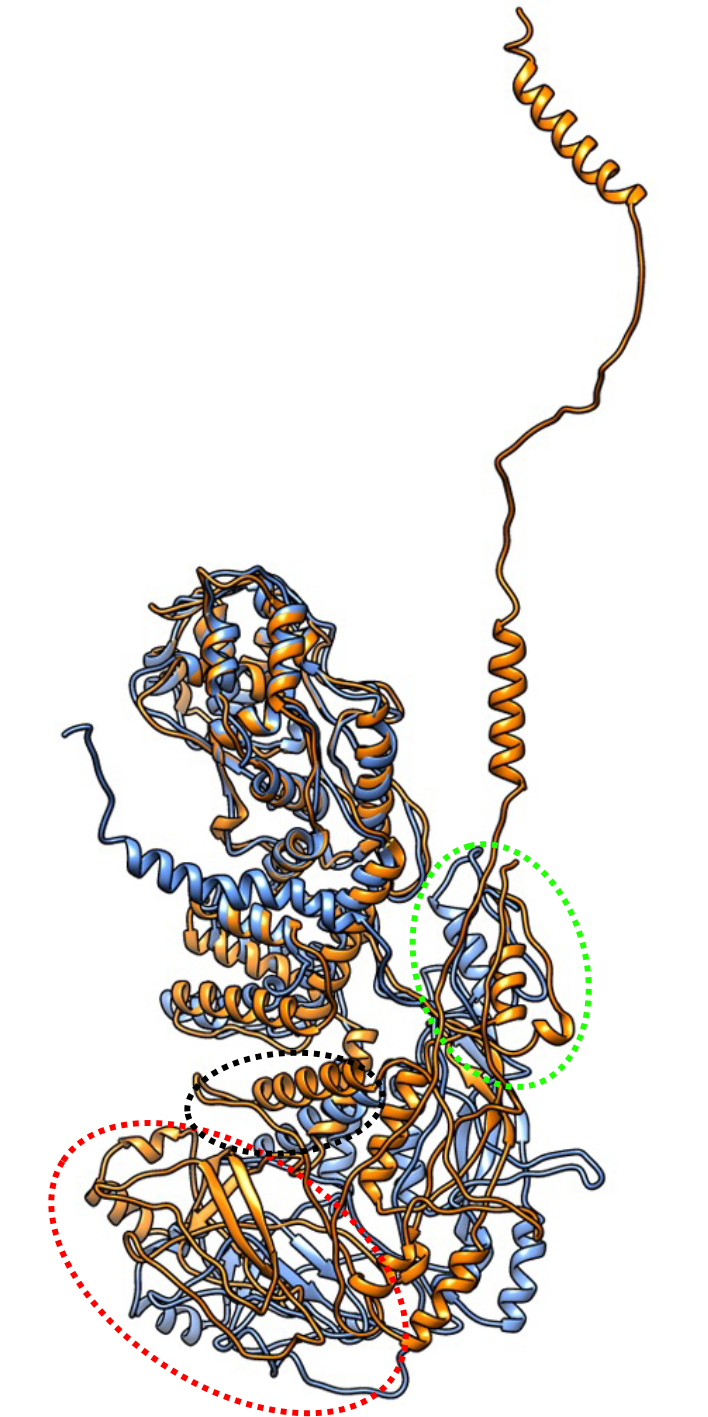

Supplement: S6 Fig — The atomic models for VP3A (cornflower blue) and VP3B (orange) are shown overlaid. There is considerable difference between the VP3A and VP3B subunits. Specifically, the biggest differences are at the distal edge of the dimerization domain (red circle), in the carapace domain (green circle) and on the inner surface of the carapace domain (black circle), and the extended N-terminus of VP3B. (TIF) [file ppat.1011341.s010.tif]

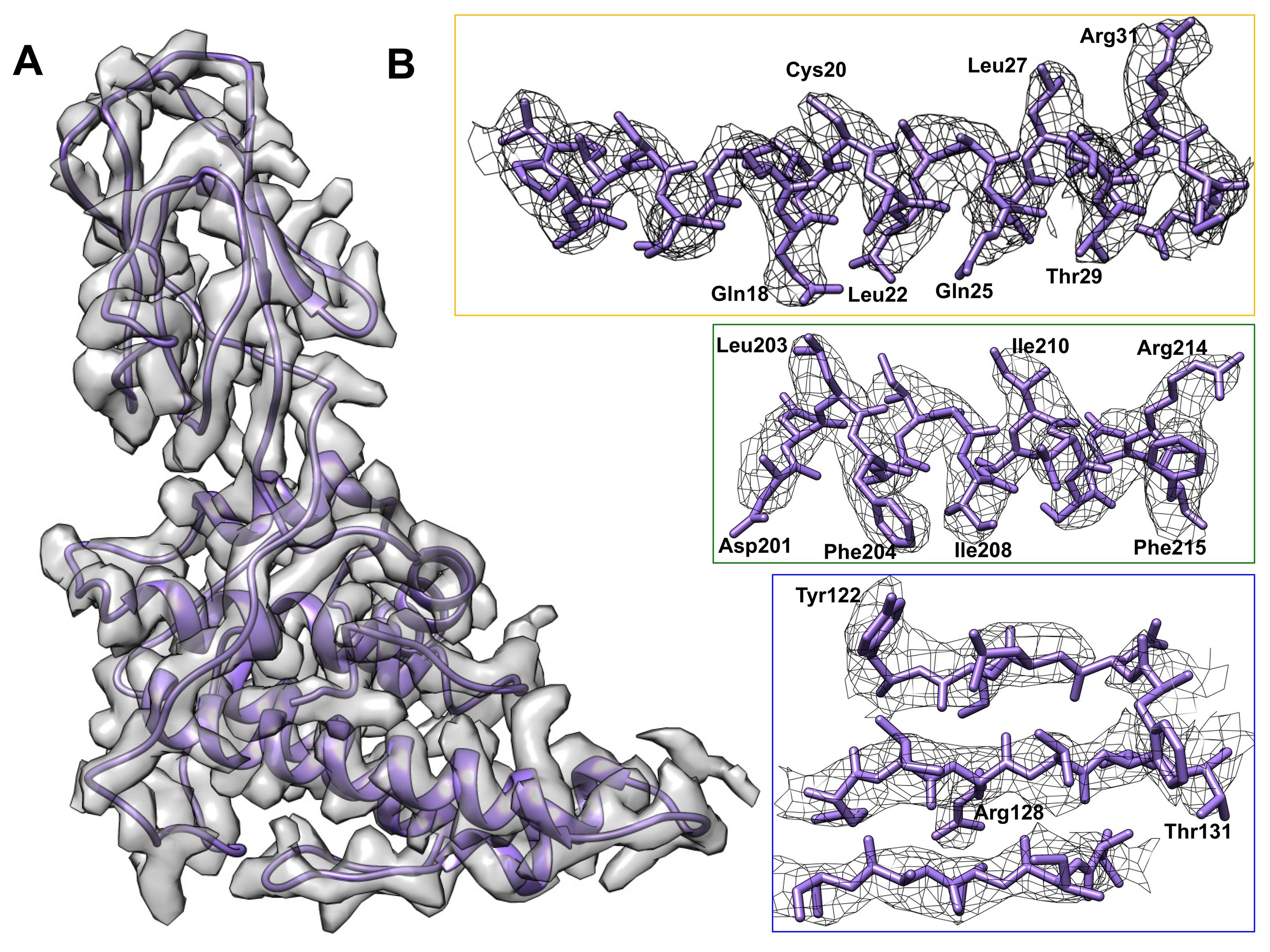

Supplement: S7 Fig — (A) Density map (gray) of VP12 superimposed with its atomic model (medium purple). (B) Zoom-in regions of the density map (black nets) superimposed with atomic model (medium purple), demonstrating the quality of cryo-EM map VP12 and that of the refined atomic model. (TIF) [file ppat.1011341.s011.tif]

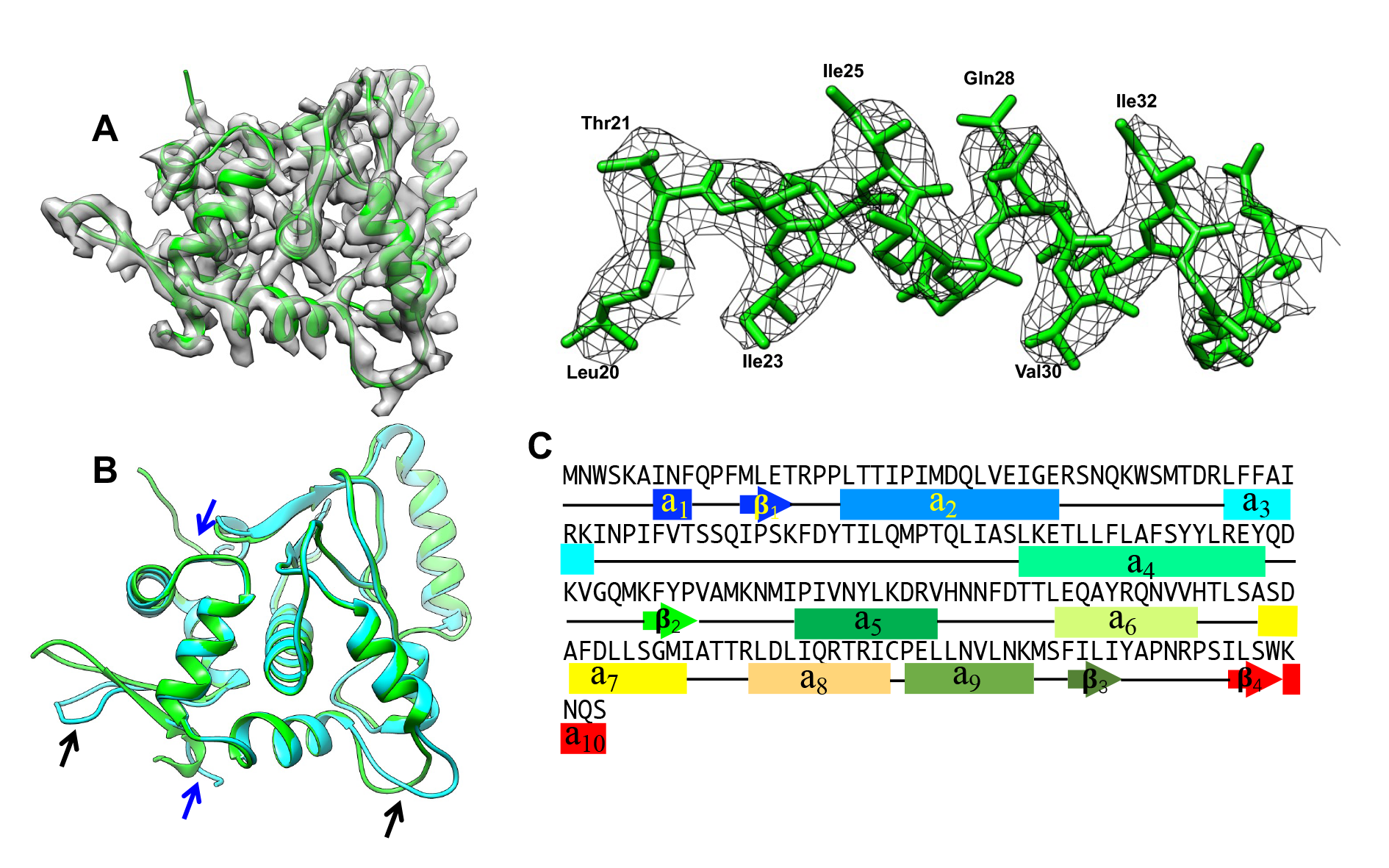

Supplement: S8 Fig — (A) Density map (gray) of VP11 superimposed with its atomic model (green). Right panel shows that zoom-in regions of the density map superimposed with atomic model, demonstrating the quality of cryo-EM map VP11 and that of the refined atomic model. (B) Alignment of VP11A and VP11B (cyan) reveals few conformation differences between them. The main differences are at the termini (blue arrows) and two peripheral loops (black arrows). (C) The sequence and secondary structural elements are indicated. The color schemes for secondary structure are the same as the model in Fig 4D. (TIF) [file ppat.1011341.s012.tif]

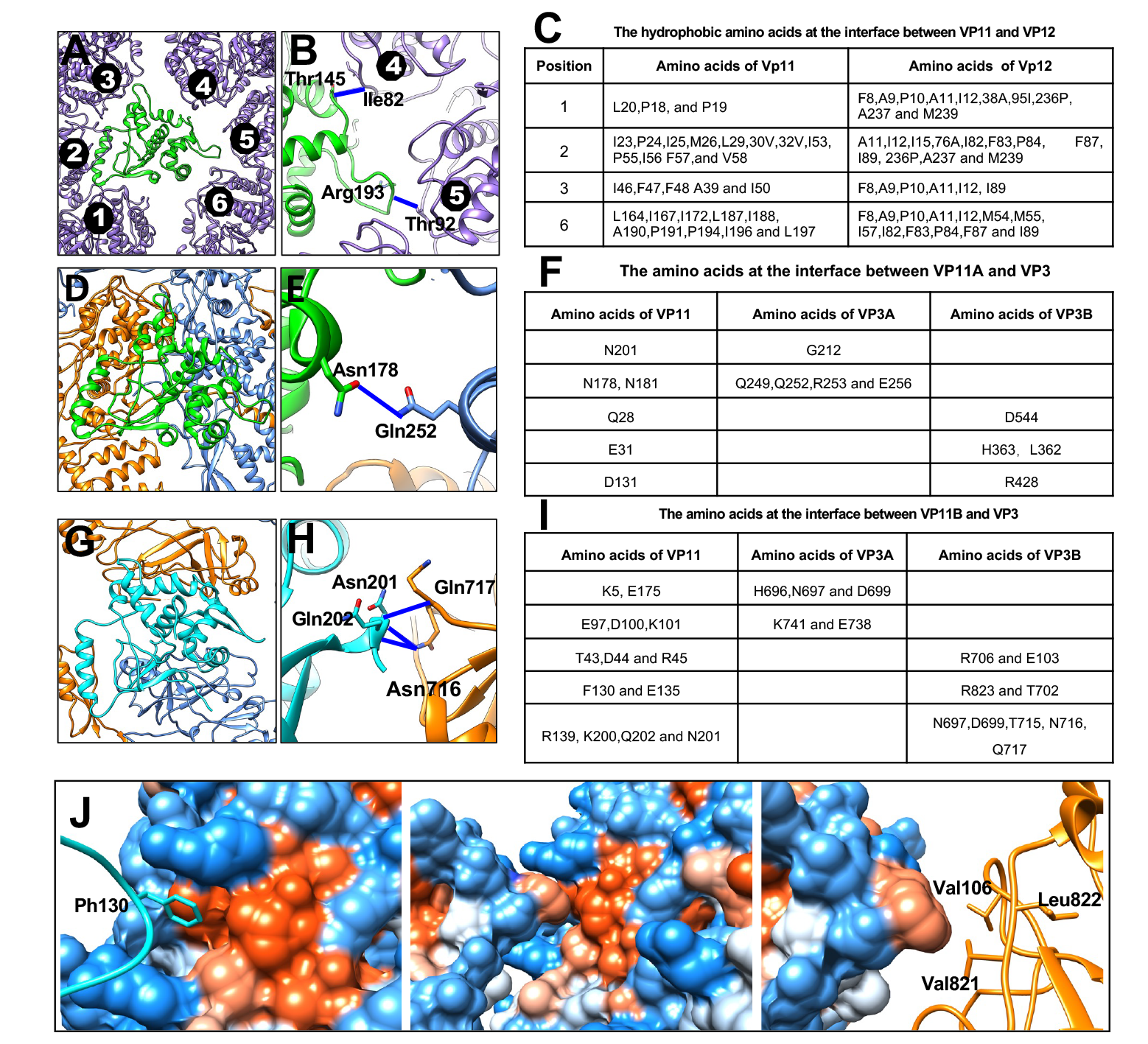

Supplement: S9 Fig — (A) VP11A interacts with the surrounding VP12s. (B) The hydrogen bonds between the VP11A and two VP12 proteins. (C) The interacting amino acids at the interface between VP11 and VP12 are indicated. (D) VP11A also interacts with the VP3s. (E) The hydrogen bond between the VP11A and VP3A. (F) The interacting amino acids at the interface between VP11A and VP3 are indicated. (G) VP11B clamps the two neighbor VP3 pentamers together. (H) The hydrogen bond between the VP11B and VP3B. (I) The interacting amino acids at the interface between VP11B and VP3 are listed. (J) The hydrophobic interactions between VP11B and VP3B, made by Phe130 in VP11 and Val106, Val 821, Leu822 in VP3. The model of VP11B is colored with cyan, while the model of VP3B is colored with orange. (TIF) [file ppat.1011341.s013.tif]

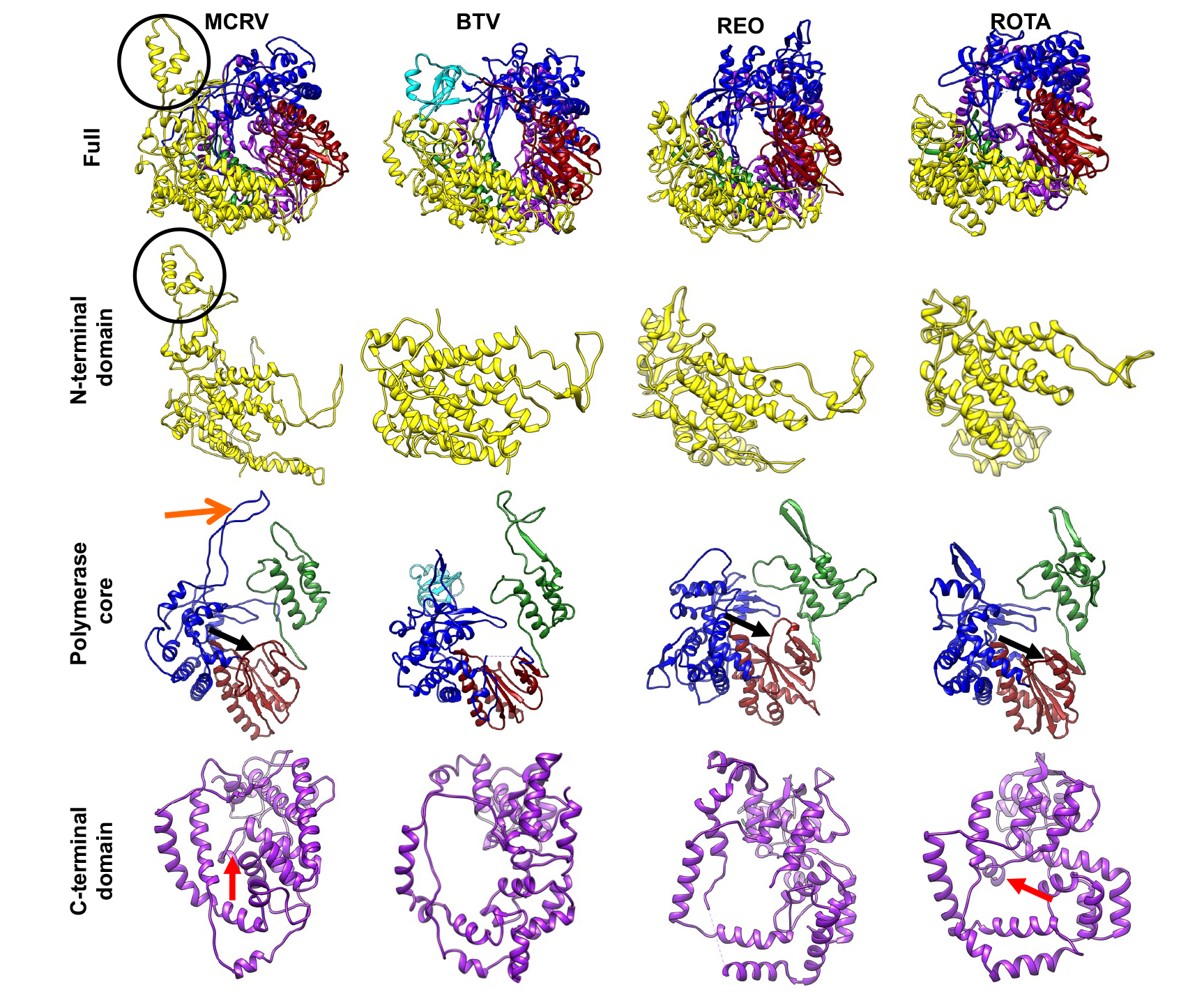

Supplement: S10 Fig — The color scheme is identical to that in Fig 5. The structure inside the black circle is the unique protrusion of the N-domain in MCRV. Black arrows indicate the priming loop; red arrows indicate the C-terminal plug. The orange arrow indicates the unique long loop blocking the gap between the thumb and finger subdomain of MCRV RdRp. The unique “fingernail motif” of bluetongue virus is colored in cyan. (TIF) [file ppat.1011341.s014.tif]

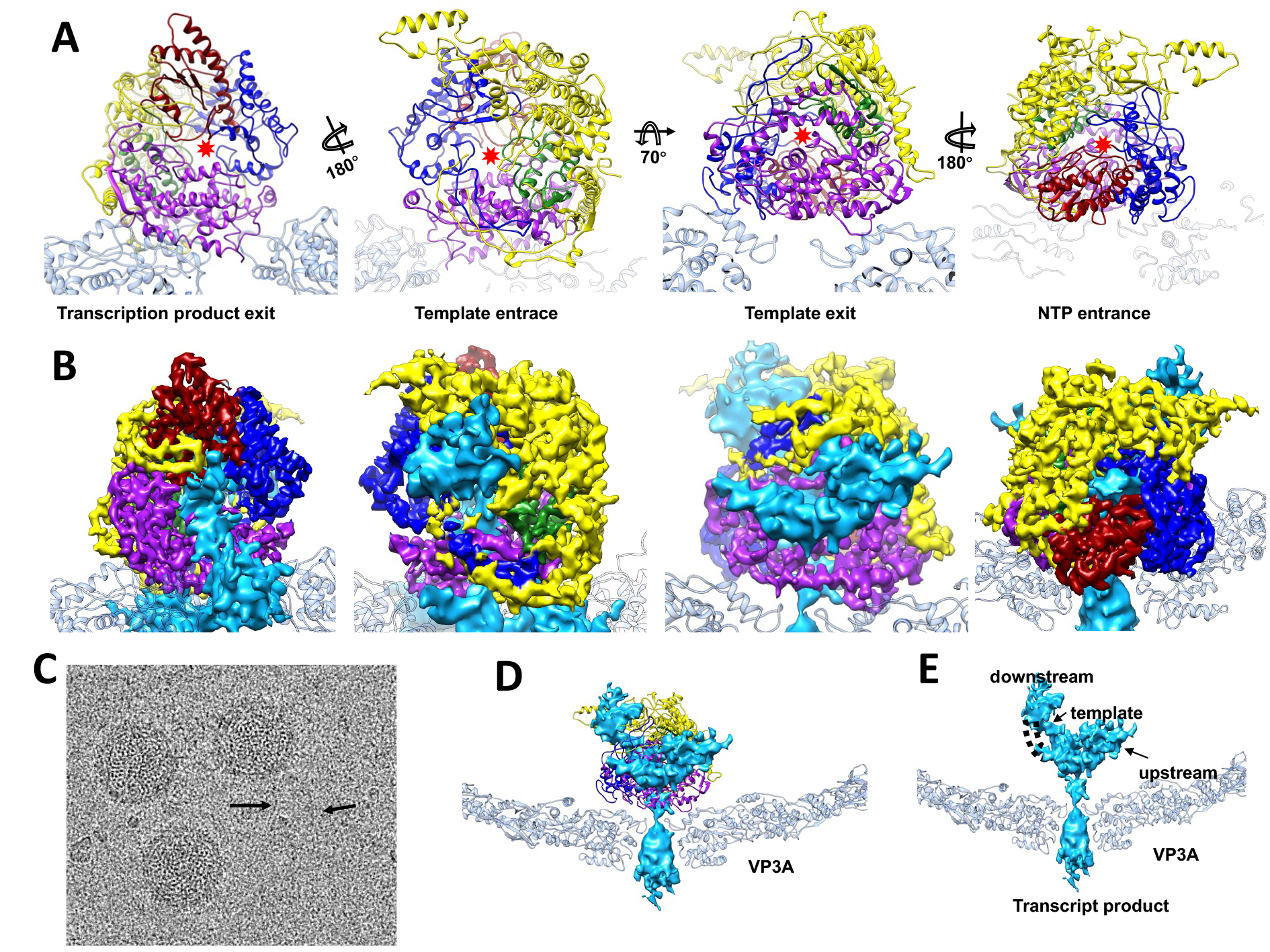

Supplement: S11 Fig — (A) indicates the four tunnels of RdRp. The asterisks indicate the positions of tunnels. The color schemes of RdRp and capsid are same as that in Figs 1 and 5. (B) indicates the RdRp map together with the extra densities (colored with light blue) in tMCRV. The orientation of each image view, together with the color schemes of RdRp and capsid are the same as that in A. (C) shows a typical cryo-EM image of tMCRV particles. Black arrows show the RNA strands. (D) The difference map (light blue) shows the extra density in the center of the RdRp. The color scheme of the RdRp model is same as that of Fig 5. The inner capsid position is shown by partial models of VP3A. (E), The extra density has features concordant with downstream dsRNA, the ssRNA template, the upstream dsRNA together with the noncontinuous non-template single strand RNA, and the transcript product. The density of non-template ssRNA is not continuous and is indicated by dot lines. The difference map is shown at a contour level of roughly 8.3. (TIF) [file ppat.1011341.s015.tif]

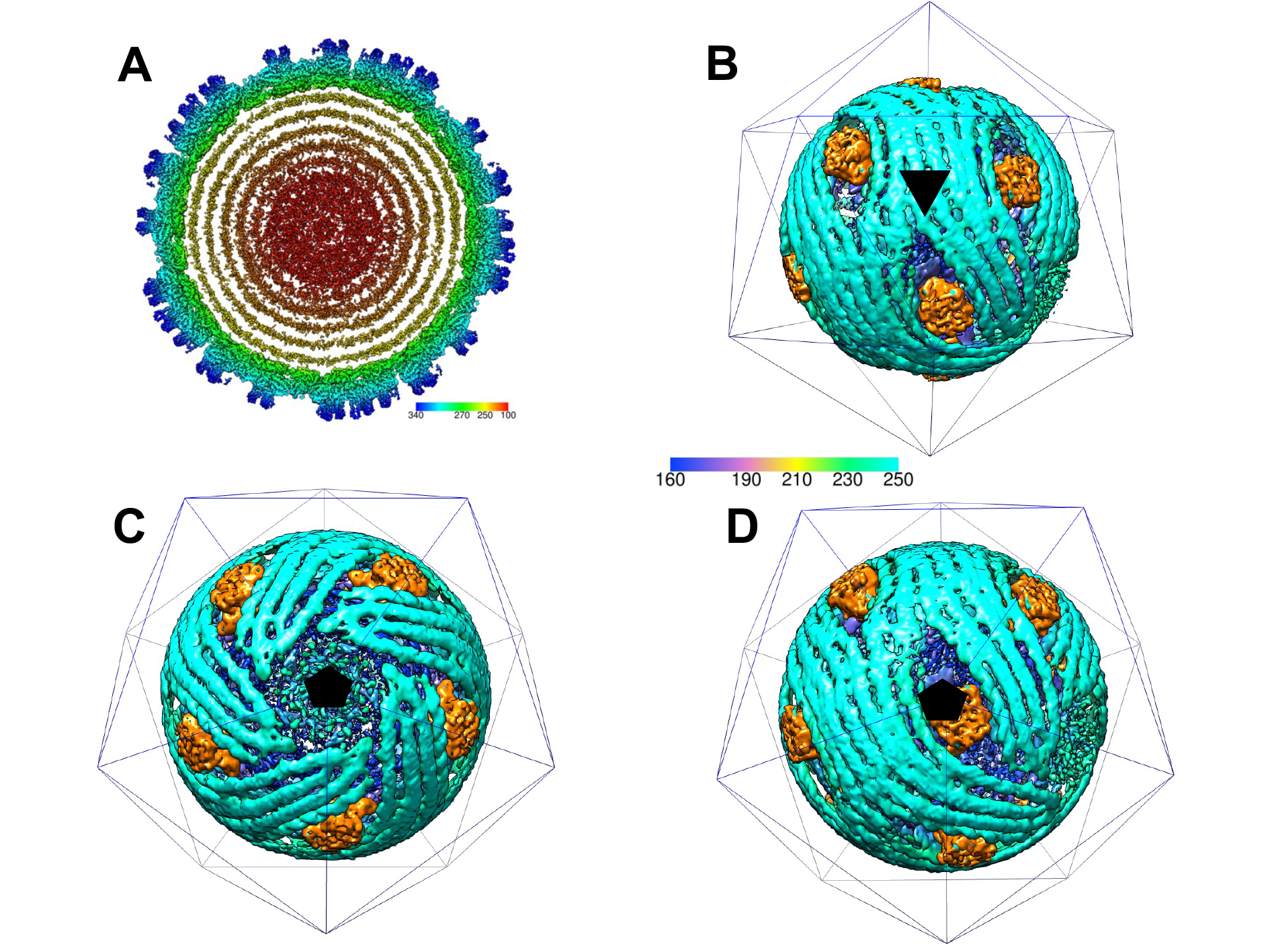

Supplement: S12 Fig — Density maps without the two capsid shells viewed along the 5-fold axis reveal that there are two kinds of icosahedral 5-fold vertices (A, B). Two of the twelve vertices have less density around the 5-fold axes (A), while at the remaining 10 vertices there is obvious RdRp density (orange) slightly offset from the 5-fold axes (B). Panels (C) and (D) show the maps along the 3- and 2-fold axis, respectively. 5-, 3-, and 2-fold axes are marked in pentagon, triangle and ellipse respectively. The map, except for the densities of polymerase, is radially colored. (TIF) [file ppat.1011341.s016.tif]

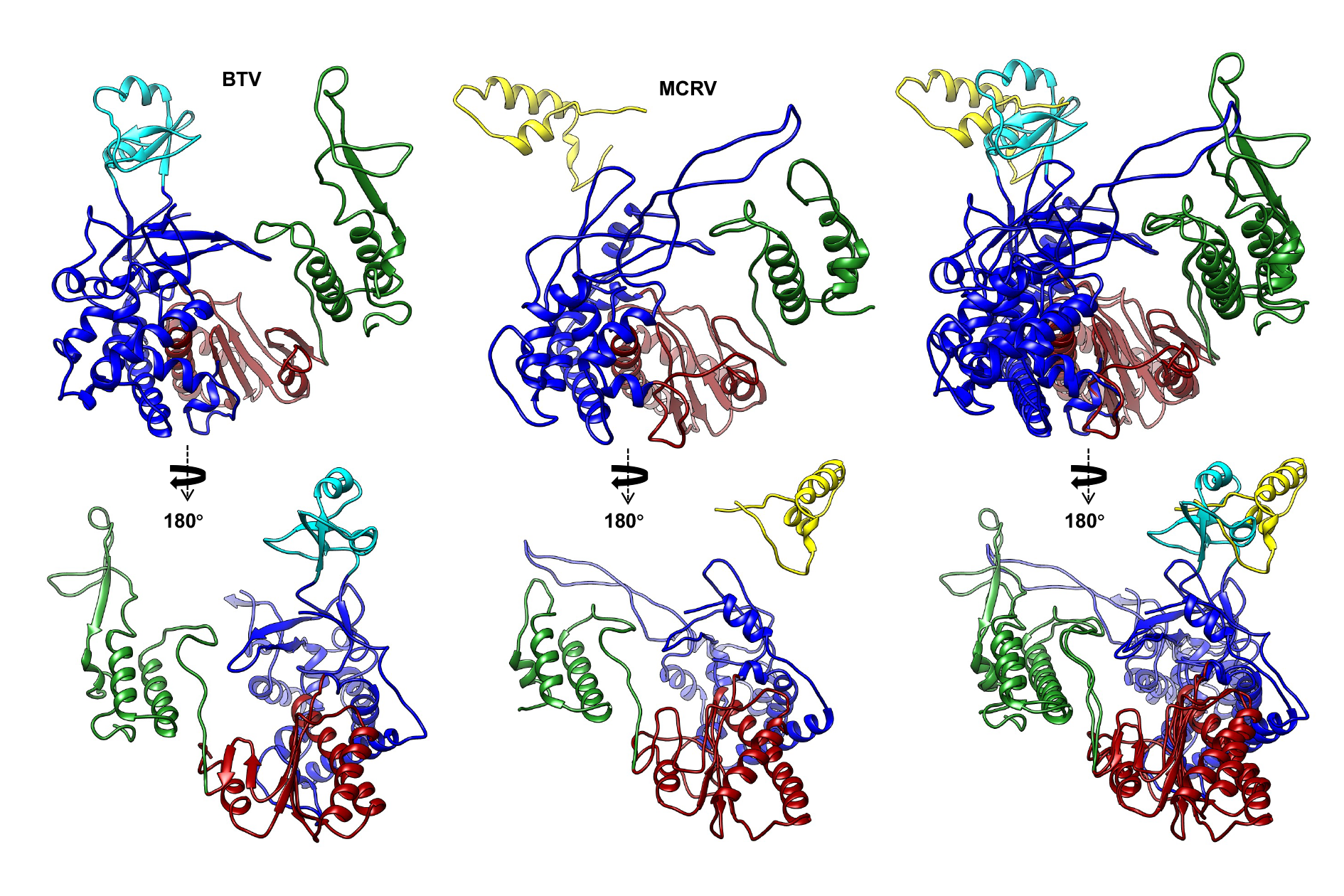

Supplement: S13 Fig — The color scheme is identical to that in S9 Fig. The left column and middle column are the polymerase of Bluetongue and MCRV respectively. The right column displays the alignment of tow polymerases. The unique protrusion (yellow) of MCRV’s N-terminal domain is displayed. The alignment of the two RdRps reveals that the unique protrusion’s position is close to that of the fingerail motif of bluetongue virus RdRp. (TIF) [file ppat.1011341.s017.tif]
